# Association of body mass index with mortality of sepsis or septic shock: an updated meta-analysis

**DOI:** 10.1186/s40560-023-00677-0

**Published:** 2023-07-03

**Authors:** Le Bai, Jingyi Huang, Dan Wang, Dongwei Zhu, Qi Zhao, Tingyuan Li, Xianmei Zhou, Yong Xu

**Affiliations:** 1 Affiliated Hospital of Nanjing University of Chinese Medicine, Nanjing, 210029 People’s Republic of China; 2 Baoshan Branch, Shuguang Hospital Affiliated to Shanghai University of Traditional Chinese Medicine, Shanghai, People’s Republic of China; 3 Department of Respiratory Medicine, Jiangsu Province Hospital of Chinese Medicine, 155 Hanzhong Road, Nanjing, 210029 Jiangsu Province People’s Republic of China; 4 School of Chinese Medicine, School of Integrated Chinese and Western Medicine, Nanjing University of Chinese Medicine, 138 Xianlin Road, Nanjing, 210029 Jiangsu Province People’s Republic of China

**Keywords:** Sepsis, Mortality, Body mass index, Obesity, Meta-analysis

## Abstract

**Background:**

The effects of body mass index (BMI) on mortality of sepsis remain unknown, since previous meta-analyses have reported conflicting results. Several observational studies published recently have provided new evidence. Thus, we performed this updated meta-analysis.

**Methods:**

PubMed, Embase, Web of Science, and Cochran Library were searched for articles published before February 10, 2023. Observational studies that assessed the association of BMIs with mortality of sepsis patients aged > 18 years were selected. We excluded studies of which data were unavailable for quantitative synthesis. Odds ratios (OR) with 95% confidence interval (CI) were the effect measure, which were combined using fixed-effect or random-effect models. The Newcastle–Ottawa Scale was applied for quality assessment. Subgroups analyses were conducted according to potential confounders.

**Results:**

Fifteen studies (105,159 patients) were included in the overall analysis, which indicated that overweight and obese BMIs were associated with lower mortality (OR: 0.79, 95% CI 0.70–0.88 and OR: 0.74, 95% CI 0.67–0.82, respectively). The association was not significant in patients aged ≤ 50 years (OR: 0.89, 95% CI 0.68–1.14 and OR: 0.77, 95% CI 0.50–1.18, respectively). In addition, the relationship between morbidly obesity and mortality was not significant (OR: 0.91, 95% CI 0.62–1.32).

**Conclusions:**

Overweight and obese BMIs (25.0–39.9 kg/m^2^) are associated with reduced mortality of patients with sepsis or septic shock, although such survival advantage was not found in all crowds.

*Trial registration* The protocol of this study was registered in PROSPERO (registration number CRD42023399559).

**Supplementary Information:**

The online version contains supplementary material available at 10.1186/s40560-023-00677-0.

## Background

Sepsis is characterized clinically by life-threatening organ dysfunction, resulting from a dysregulated host response to severe systemic infection [1]. In 2017, the sepsis-related deaths were estimated 11.0 million, accounting for approximately 20% of global deaths, and it has become the leading cause of in-hospital mortality [2].

Obesity was reported to be associated with increased risk of sepsis [3], and more than one-quarter of adults admitted to intensive care units (ICU) have obese or overweight body mass indexes (BMI). In animal models, it was demonstrated that obesity could enhance sepsis-induced organ injury [4–6]. Thus, it is reasonable to speculate that obese BMI could worsen clinical outcomes. Surprisingly, clinical studies have reported mixed results. A systematic review revealed that obesity may decrease, increase, or not affect the survival [7]. Based on this, Pepper et al. undertook a meta-analysis and found that overweight and obese patients with sepsis were at a decreased risk of death [8]. Nevertheless, another meta-analysis conducted by Wang et al. suggested that overweight, rather than obesity or morbid obesity could reduce the mortality of patients with sepsis [9].

Consecutive meta-analyses and systematic reviews have failed to reach a consensus. An obvious limitation of these previously published studies is that evidence from prospective studies was insufficient. Moreover, it was discovered that the association between obesity and outcomes of interest could be confounded [10]. However, bias analyses were not performed in prior meta-analyses due to insufficient included studies, and the issues remain unsolved till now. Therefore, we conducted an updated meta-analysis, including current available studies, to summarize the latest and most comprehensive evidence. We also performed subgroup analyses according to possible biases, intending to better clarify the association between obesity and mortality of sepsis.

## Methods

We undertook and reported the meta-analysis and systematic review in accordance with Meta-analysis of Observational Studies in Epidemiology (MOOSE) proposal [11]. Please see the MOOSE checklist in Additional file 1.

Two researchers (Le Bai and Jingyi Huang) were in charge of literature retrieval, data extraction, and quality assessment of eligible studies independently. A third researcher (Dan Wang) would be consulted if the disputation could not be resolved by discussion.

### Literature search and study selection

We performed the literature search without any language restriction in the following electronic databases: PubMed, Embase, Cochrane Library, and Web of Science. Articles published before February 10, 2023 were retrieved. Please see the detailed search strategy in Additional file 2.

Observational studies that evaluated the association between obesity and mortality of patients diagnosed with sepsis or septic shock were included. The outcomes should be short-term (˂ 30 days) mortality. BMI was utilized as the measure of obesity in our study, which was calculated as the following formula: BMI = weight (kg)/height × height (m^2^). The control groups were patients with normal BMIs (18.5–24.9 kg/m^2^) while the exposure groups consisted of patients with abnormal BMIs. The following studies were excluded: (1) studies that recruited patients aged ˂ 18 years; (2) studies with incomplete data for the systematic review and meta-analysis; (3) studies that did not select patients with normal weight as control groups; (4) studies in which BMI was considered as a continuous variable rather than a categorical variable; (5) studies published in the forms of letter, comment or conference abstract. In addition, we selected odds ratio (OR) with 95% confidence interval (CI) as the effect measure, since most individual studies have reported ORs. Thus, studies that reported relative risk (RR) or hazard ratio (HR) were not included in the quantitative synthesis in case of potential bias.

### Data extraction and risk of bias assessment

Extracted data included author, study design, country or region, publication year, population characteristics of included study, diagnostic criteria of sepsis (or septic shock), definition of exposure and control groups, outcome, and adjusted covariates in each study.

The Newcastle–Ottawa Scale (NOS) was employed for quality evaluation of eligible studies [12]. The details of the scale and standards of grading are provided in Additional file 3. It was designed to evaluate the quality of non-randomized controlled trials and has been the most widely used tool for observational studies in systematic reviews [13]. The scale consisted of three parts, which were aimed to evaluated the risk of selection bias (comparability between control and exposure groups), information bias (ascertainment of exposure and outcome), and confounding bias.

Several other factors (e.g., age, study design) were also reported to be clinically relevant to the mortality of sepsis [8, 10], which could confound the real effects of obesity on mortality of sepsis. These issues have not been addressed in previous meta-analyses [8, 9]. Thus, in the present meta-analysis, subgroup analyses were undertaken to further investigate whether obesity and sepsis are truly associated. In addition, publication bias was evaluated using the funnel plot.

### Data synthesis and statistical analysis

In the quantitative synthesis, comparisons were made between patients with normal BMIs versus those with abnormal BMIs. OR with 95% CI was selected as the effect measure. Forest plots were employed to show the results for effects of obesity on mortality of sepsis. I-squared (*I*^*2*^) statistics was used for assessment of the heterogeneity. Random-effect model was selected if the heterogeneity was high (*I*^*2*^ > 50%). Otherwise, the fixed-effect model would be selected. We conducted all statistical analyses using Stata 15.0 (Stata Corp, College Station, Texas, USA).

## Results

### Study selection and characteristics of eligible studies

**Fig. 1:**
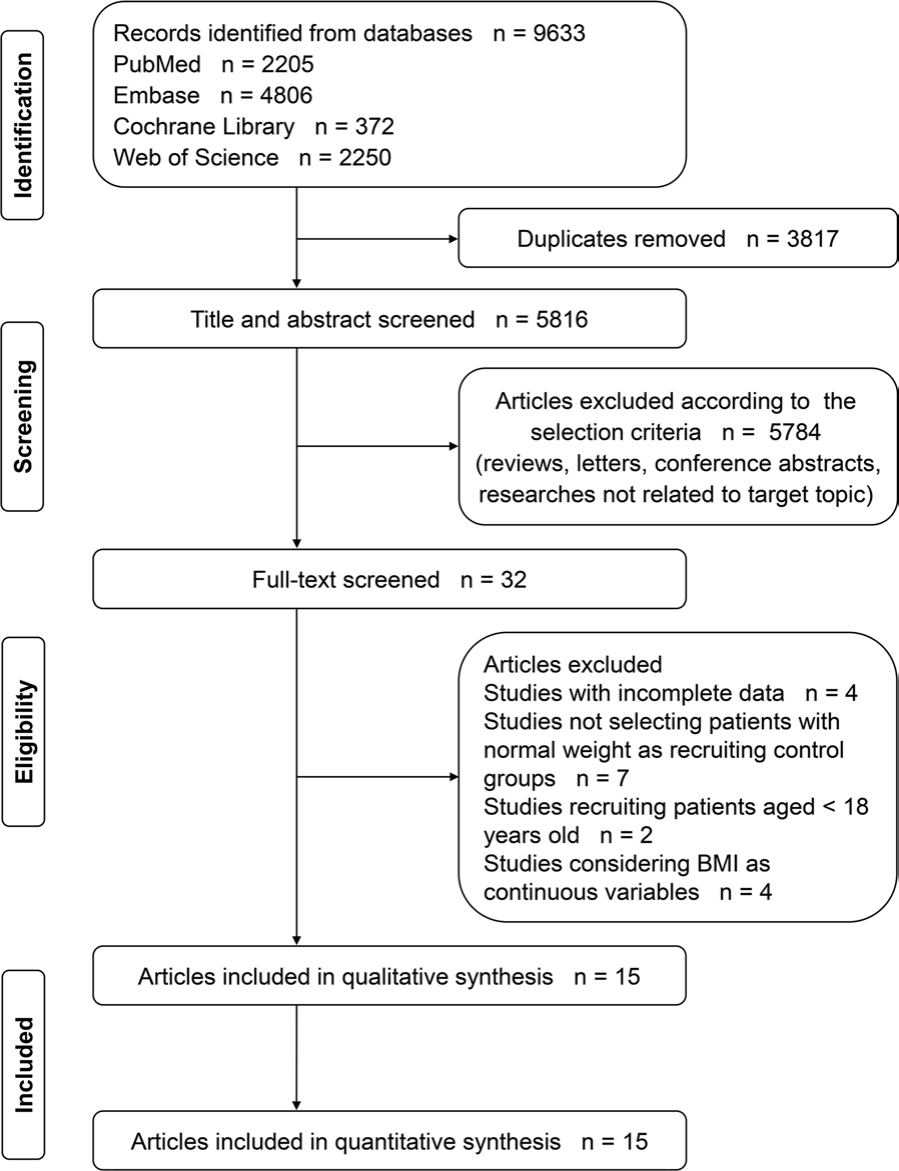
The flow chart of study selection

A total of 9633 records were yielded from all databases, according to the initial retrieval. After removing the duplicates, we screened titles and abstracts of rest literature, and obtained 32 potentially eligible articles. We then reviewed the full text and included 15 studies in the quantitative synthesis. The process of study selection is presented in Fig. 1.

Three prospective studies [14–16] and 12 retrospective studies [17–28] were included in the present meta-analysis. Ten studies were from the United States [17–26], two were from Korea [14, 15], one was from Austria [28], and the other two were multicenter studies [16, 27]. These studies were published between 2008 and 2023. The subjects were patients with sepsis, severe sepsis or septic shock, and the sample size ranged from 301 to 55,038. In most studies (12 of all) [14–17, 19, 20, 22–25, 27, 28], the diagnoses were based on the criteria defined on the three international conferences (i.e., Sepsis 1.0, Sepsis 2.0, and Sepsis 3.0 criteria) [1, 29, 30]. In two studies [18, 21], sepsis and septic shock were diagnosed according to the diagnostic codes in databases, and the diagnostic criteria were not available in one study[26]. The outcomes included ICU, in-hospital, and 28-day (or 30-day) mortality. In addition, multiple covariates were adjusted in each study and the details are summarized in Table 1.

**Table 1:** Characteristics of eligible studies

Author	Study design	Publish year	Region	Age	Study population	Sample size	Diagnosis of sepsis	BMI category, kg/m^2^	Outcome	Adjusted covariates
Wurzinger et al. [28]	Retrospective cohort study	2010	Austria	≥ 18 years	Patients with septic shock	301	Sepsis 2.0	Underweight (< 18.5); normal weight (18.5–24.9, ref); overweight (25.0–29.9); obesity (≥ 30.0)	ICU mortality	Admission year, sex, age, presence of heart disease or chronic renal insufficiency, the number of pre-morbidities, origin of sepsis and the simplified acute physiology score II
Pepper et al. [20]	Retrospective cohort study	2019	United States	≥ 20 years	Patients with sepsis	55,038	Sepsis 3.0	Underweight (< 18.5); normal weight (18.5–24.9, ref); overweight (25.0–29.9); obesity Class-I (30.0–34.9); obesity Class-II (35.0–39.9); obesity Class-III (≥ 40.0)	Short-term mortality	Variables which are considered clinically relevant (details are NA)
Tay-Lasso et al. [17]	Retrospective cohort study	2022	United States	≥ 18 years	Patients with severe sepsis after trauma	1,246	Sepsis 2.0 (sepsis + organ dysfunction, hypotension, or hypoperfusion to 1 or more organs)	Underweight (< 18.5); normal weight (18.5–24.9, ref); overweight (25.0–29.9); obesity (30.0–34.9); severe obesity (35.0–39.9); morbid obesity (≥ 40.0)	In-hospital mortality	Age, alcohol use, hypertension, congestive heart failure, diabetes mellitus, injury severity score, respiratory rate, pulse rate, systolic blood pressure, intensive care unit days, Glasgow coma scale
Kuperman et al. [26]	Retrospective cohort study	2013	United States	≥ 18 years	Patients with sepsis	792	NA	Underweight (< 18.5); normal weight (18.5–24.9, ref); overweight (25.0–29.9); obesity (30.0–39.9); morbid obesity (40.0–49.9)	In-hospital mortality	Age, race, gender, length of stay, comorbidities, APACHE II score
Prescott et al. [24]	Retrospective cohort study	2014	United States	Older patients (> 50 years)	Patients with severe sepsis	1,404	Sepsis 2.0	Normal weight (18.5–24.9, ref); overweight (25.0–29.9); obesity (30.0–34.9); morbid obesity (≥ 40.0)	In-hospital, 90-day, and 1-year mortality	Age, race, gender, marital status, wealth, acute organ dysfunctions, mechanical ventilation, comorbidities, baseline cognitive status, functional limitations
Sakr et al. [16]	Prospective cohort study	2008	European multicenter	≥ 15 years	Patients with sepsis	2,878	Sepsis 1.0	Underweight (< 18.5); normal weight (18.5–24.9, ref); overweight (25.0–29.9); obesity (30.0–39.9); morbid obesity (≥ 40.0)	In-hospital, 60-day mortality	Age, gender, comorbidities, SAPS II score and SOFA score, the type of admission, mechanical ventilation, renal replacement therapy
Lin et al. [19]	Retrospective cohort study	2020	United States	≥ 18 years	Patients with sepsis	7,967	Sepsis 3.0	Underweight (< 18.5); normal weight (18.5–24.9, ref); overweight (25.0–29.9); obesity (≥ 30.0)	28-day mortality	Age, sex, SOFA, mechanical ventilation, renal replacement therapy, comorbidities, alcohol abuse, drug abuse, depression
Danninger et al. [18]	Retrospective cohort study	2022	United States	NA	Patients with sepsis	16,612	Diagnostic code in the eICU Collaborative Research database	Underweight (< 18.5); normal weight (18.5–24.9, ref); overweight (25.0–29.9); obesity (≥ 30.0)	ICU mortality	Age, sex, creatinine concentration, ethnics, heart rate, infection focus, lactate concentration, SOFA score, mechanical ventilation, vasopressor use
Gaulton et al. [23]	Retrospective cohort study	2014	United States	≥ 18 years	Patients with severe sepsis	1,191	Sepsis 2.0	Underweight (< 18.5); normal weight (18.5–24.9, ref); overweight (25.0–29.9); obesity (30.0–39.9); morbid obesity (≥ 40.0)	28-day mortality	Age, APACHE II, intubation, oncology service, initiation of early goal directed therapy, creatinine clearance
Gaulton et al. [25]	Retrospective cohort study	2015	United States	≥ 18 years	Patients with sepsis	1,779	Similar to sepsis 1.0*	Non-obesity (18.5–29.9); obesity (≥ 30.0)	28-day mortality	Age, gender, race, year and hospital of admission, length of stay, drug use, positive blood cultures, severity, comorbidities
Arabi et al. [27]	Retrospective cohort study	2013	Multicenter	Adult patients	Patients with septic shock	2,882	Sepsis 1.0	Underweight (< 18.5); normal weight (18.5–24.9, ref); overweight (25.0–29.9); obesity (30.0–39.9); morbid obesity (≥ 40.0)	In-hospital mortality	Age, gender, mechanical ventilation, APACHE II score, chronic co-morbidities, infections, creatinine clearance
Chae et al. [15]	Prospective cohort study	2013	Korea	≥ 18 years	Patients with severe sepsis or septic shock	770	Sepsis 1.0	Underweight (< 18.5); normal weight (18.5–24.9, ref); obese (≥ 25.0)	In-hospital mortality	Age, gender, comorbidities, infection focus, SOFA score, serum lactate
Li et al. [21]	Retrospective cohort study	2019	United States	≥ 18 years	Patients with sepsis, severe sepsis or septic shock	5,563	Diagnostic code in the MIMIC-III database	Underweight (< 18.5); normal weight (18.5–24.9, ref); overweight (25.0–29.9); obesity (≥ 30.0)	30-day, and 1-year mortality	Age, gender, race, marriage status, admission ICU type, MAP, SAPS II, SOFA, surgery
Juarez et al. [22]	Retrospective cohort study	2019	United States	18–89 years	Patients with sepsis or septic shock	312	Sepsis 3.0	Underweight (< 18.5); normal weight (18.5–24.9, ref); overweight (25.0–29.9); obesity Class-I (30.0–34.9); obesity Class-II (35.0–39.9); obesity Class-III (≥ 40.0)	In-hospital mortality	Age, gender, comorbidities, APACHE II
Yeo et al. [14]	Prospective cohort study	2023	Korea	≥ 18 years	Patients with sepsis	6424	Sepsis 3.0	Underweight (< 18.5); normal weight (18.5–24.9, ref); overweight (25.0–29.9); obesity (≥ 30.0)	In-hospital mortality	Age, sex, comorbidities, SOFA score, presence of septic shock, site of infection, type of infection

*BMI* body mass index, *Ref* reference, *NA* not available, *APACHE II* acute physiology and chronic health evaluation II, *SOFA* sequential organ failure assessment, *MAP* mean arterial pressure, *SAPS II* simplified acute physiology score II

*About 94.5% positive predictive value for sepsis 1.0

### Risk of bias assessment

According to the NOS scale, all studies are considered as high quality. Details are provided in Additional file 4. We also focused on the potential confounders as follows: (1) age; Abbate et al. found that the association between obesity and mortality of sepsis was significant in patients aged 50–89 years but not in those aged 20–49 years [10]; (2) study design; retrospective cohort studies are easily subject to bias (e.g., recall bias, data integrity) and prospective studies could better clarify the relationship between obesity and outcomes of interest; (3) diagnostic criteria of sepsis; the specificity and sensitivity of different criteria may be different; (4) severity of sepsis; concerns about hypoventilation and insufficient care in obese patients on general wards may lead to ICU admission of patients with mild infection [8].

### Meta-analysis and bias analysis

Fifteen studies [14–28] containing 105,159 patients were included in the meta-analysis. The reference groups were patients with normal BMIs (18.5–24.9 kg/m^2^) while the exposure groups were patients with underweight (< 18.5 kg/m^2^), overweight (25.0–29.9 kg/m^2^), obese (30.0–39.9 kg/m^2^), and morbidly obese BMIs (≥ 40.0 kg/m^2^).

**Fig. 2:**
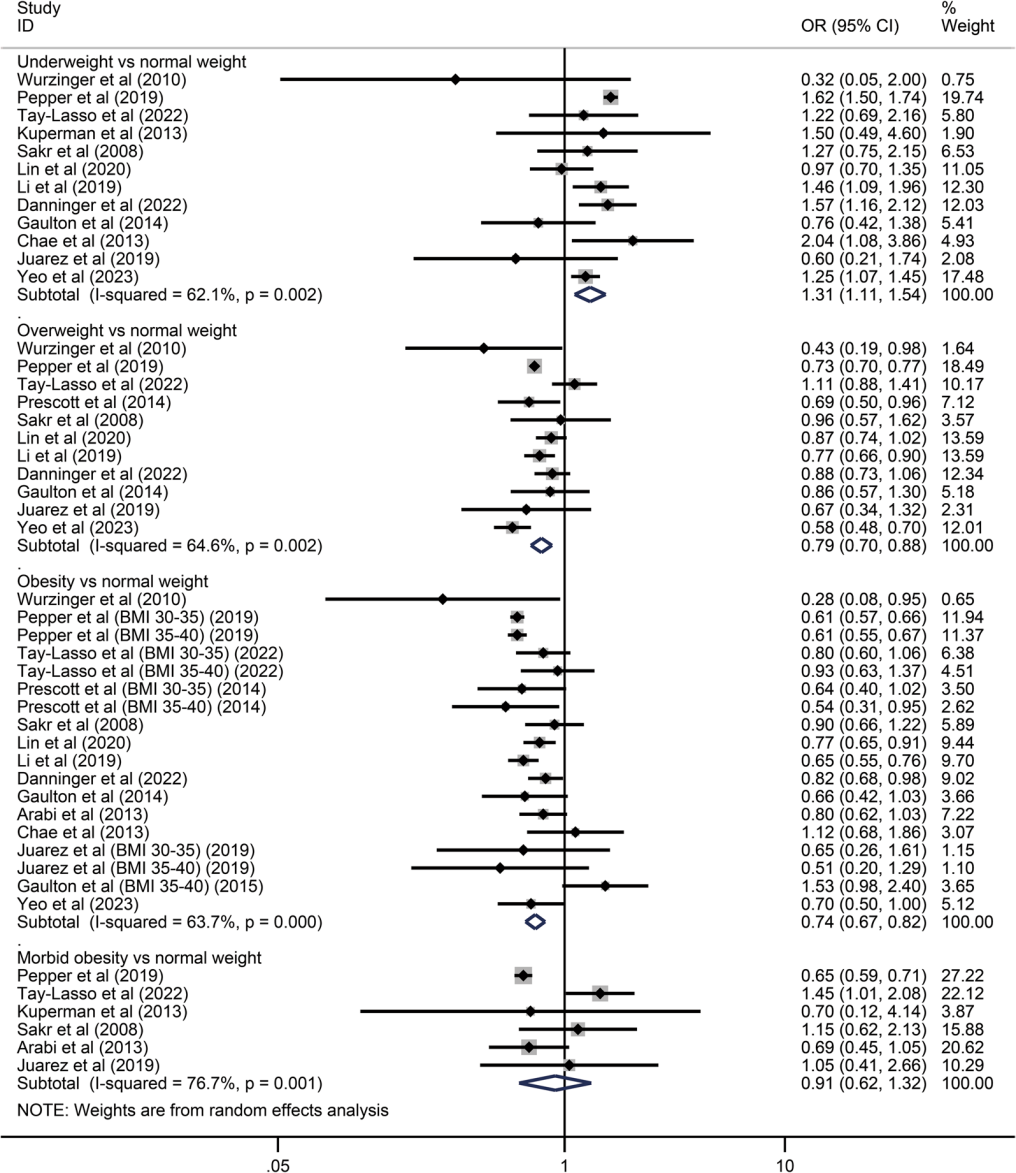
Individual and pooled results of the association of underweight, overweight, obese, and morbidly obese BMIs with mortality in patients with sepsis

The primary analysis indicated that overweight and obesity were associated with decreased mortality (OR: 0.79, 95% CI 0.70–0.88 and OR: 0.74, 95% CI 0.67–0.82, respectively) while underweight BMIs were associated with increased mortality (OR: 1.31, 95% CI 1.11–1.54). The association between morbid obesity and mortality was not significant (OR: 0.91, 95% CI 0.62–1.32). Please see Fig. 2.

We then conducted subgroup analyses for patients with underweight, overweight and obese BMIs, according to age, study design, diagnosis and severity of sepsis. Since only six studies [16, 17, 20, 22, 26, 27] reported results including a measure between morbid obesity and mortality, secondary analyses for these patients were not performed. Results of subgroup analyses are displayed in Table 2. In patients aged > 50 years, underweight BMIs were associated with higher mortality (OR: 1.58, 95% CI 1.46–1.71) while overweight and obese BMIs were associated with lower mortality (OR: 0.77, 95% CI 0.67–0.89 and OR: 0.62, 95% CI 0.59–0.65, respectively). In patients aged ≤ 50 years, underweight BMIs were associated with higher mortality (OR: 1.72, 95% CI 1.35–2.19) while the association of overweight and obese BMIs with mortality was not significant (OR: 0.89, 95% CI 0.68–1.14 and OR: 0.77, 95% CI 0.50–1.18, respectively). Please see Additional file 5: Fig. S1A–F. In retrospective studies [17–28], underweight BMIs were associated with increased mortality (OR: 1.26, 95% CI 1.01–1.56) while overweight and obese BMIs were associated with decreased mortality (OR: 0.81, 95% CI 0.73–0.91 and OR: 0.72, 95% CI 0.64–0.80, respectively). In prospective studies [14–16], underweight BMIs were associated with higher mortality (OR: 1.28, 95% CI 1.11–1.47) while overweight and obese BMIs had no impact on mortality (OR: 0.70, 95% CI 0.43–1.13 and OR: 0.85, 95% CI 0.69–1.05, respectively). Please see Additional file 5: Fig. S2A–F. In studies where the diagnoses of sepsis were based on Sepsis 1.0 or 2.0 criteria [15–17, 23–25, 27, 28], underweight and overweight BMIs were not associated with mortality (OR: 1.19, 95% CI 0.89–1.58 and OR: 0.84, 95% CI 0.65–1.10, respectively) while obese BMIs were related to decreased mortality (OR: 0.83, 95% CI 0.74–0.94). In studies where sepsis was diagnosed according to Sepsis 3.0 criteria [14, 19, 20, 22], underweight did not influence the mortality (OR: 1.24, 95% CI 0.95–1.62) while overweight and obese BMIs were associated with lower mortality (OR: 0.72, 95% CI 0.62–0.84 and OR: 0.63, 95% CI 0.59–0.66, respectively). Please see Additional file 5: Fig. S3A–F. In patients with sepsis, underweight BMIs were associated with increased mortality (OR: 1.34, 95% CI 1.13–1.59) while overweight and obese BMIs were associated with decreased mortality (OR: 0.77, 95% CI 0.69–0.86 and OR: 0.72, 95% CI 0.64–0.82, respectively). In patients with severe sepsis or septic shock, underweight and overweight BMIs were not related to mortality (OR: 1.17, 95% CI 0.84–1.61 and OR: 0.80, 95% CI 0.61–1.05, respectively) while obese BMIs were associated with decreased mortality (OR: 0.79, 95% CI 0.69–0.91). Please see Additional file 5: Fig. S4A–F.

**Table 2:** Subgroup analyses according to age, study design, diagnosis and severity of sepsis

	Underweight	Overweight	Obesity
Age			
> 50 years	1.58 (1.46–1.71)	0.77 (0.67–0.89)	0.62 (0.59–0.65)
≤ 50 years	1.72 (1.35–2.19)	0.89 (0.68–1.14)	0.77 (0.50–1.18)
Study design			
Retrospective study	1.26 (1.01–1.56)	0.81 (0.73–0.91)	0.72 (0.64–0.80)
Prospective study	1.28 (1.11–1.47)	0.70 (0.43–1.13)	0.85 (0.69–1.05)
Diagnostic criteria			
Sepsis 1.0 or 2.0	1.19 (0.89–1.58)	0.84 (0.65–1.10)	0.83 (0.74–0.94)
Sepsis 3.0	1.24 (0.95–1.62)	0.72 (0.62–0.84)	0.63 (0.59–0.66)
Severity			
Patients with sepsis	1.34 (1.13–1.59)	0.77 (0.69–0.86)	0.72 (0.64–0.82)
Patients with severe sepsis or septic shock	1.17 (0.84–1.61)	0.80 (0.61–1.05)	0.79 (0.69–0.91)

**Fig. 3:**
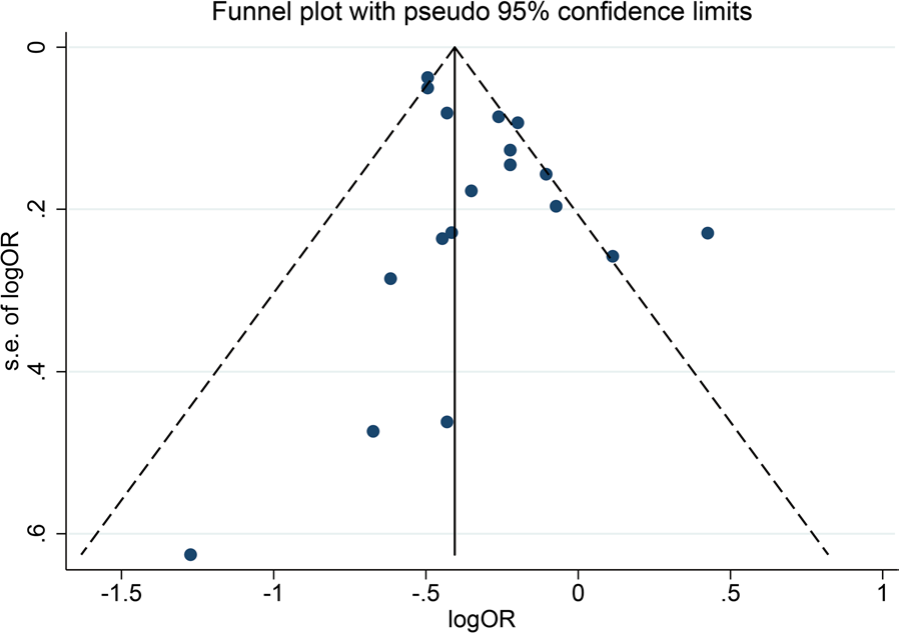
The funnel plot of the publication bias assessment for obese BMI (30.0–39.9 kg/m^2^) groups.* BMI* body mass index

In addition, the funnel plot for obese BMI groups indicated significant publication bias. Please see Fig. 3.

## Discussion

This is an updated meta-analysis, which was designed to evaluate the effects of BMI on mortality of sepsis. Consistent with Pepper et al.’s meta-analysis [8], the present one indicated that overweight and obese BMIs were associated with decreased mortality. However, we included more studies and the results were thereby more convincing with improved statistical power. On the other hand, stricter standards were adopted for study selection in our meta-analysis to control bias. In previous analyses, outcomes including in-hospital, 28-day and 60-day mortality were pooled. In comparison, we only included studies that reported short-term mortality (˂ 30 days). Meanwhile, we excluded studies that used HR as the effect measure (these studies were included in previous pooled analyses), given that HR involves a time factor which could be the possible source of bias.

Concerns were raised that significant reduction of mortality may result from methodology in studies rather than increased BMIs themselves [8]. Similarly, Robinson et al. [31] emphasized the importance of stratifying risk factors and controlling for covariates, which are conducive to describe the phenotype of sepsis survivors. Thereby, in the present meta-analysis, subgroup analyses were performed in accordance to potential confounders. Abbate et al. [10] discovered that age may modify the association of BMIs with mortality. We obtained the similar conclusion which suggested that only patients aged over 50 years could benefit from higher BMIs. Pepper et al. [8] also raised concerns about ICU admission of obese and overweight patients with mild infection, which could cause selection bias. Accordingly, we conducted secondary analyses for those diagnosed with severe sepsis or septic shock. Although overweight BMIs did not decrease the risk of death, these patients could still benefit from obese BMIs.

Pepper et al.’s [8] pooled analysis of three studies suggested that underweight had no impact on the mortality of sepsis. Our meta-analysis included 12 studies and found that underweight patients, compared with patients with normal BMIs, were at higher risk of death. Similarly, several cohort studies suggested that underweight BMIs was associated with increased mortality, compared with non-underweight BMIs [32–34]. In addition to protective effects of higher BMIs, possible pathophysiological reasons include poor nutritional status, persistent inflammation and catabolism syndrome [35]. It should be noted that the association between underweight BMIs and mortality was not significant in studies, where diagnoses of sepsis were based on the consensus reached in the several international conferences (Sepsis 3.0 or Sepsis 1.0/2.0) [1, 29, 30]. The association between overweight BMIs and mortality are subject to different diagnosis criteria as well. As yet it is unclear whether the diagnosis of sepsis confounded the association of BMI with mortality. However, specificity and sensitivity of other criteria were inferior to those of consensus criteria, and early and rapid identification of sepsis could help to improve clinical outcomes [36]. Therefore, the diagnostic criteria which are not recognized should be avoided in future studies.

Two prospective, multicenter studies (Sakr et al. [16] and Yeo et al. [14]) were included in the present meta-analysis, but the results were inconsistent with each other. On one hand, Sakr et al.’s study [16] was conducted during 2002 while Yeo et al.’s study [14] was conducted between 2019 and 2020; the management of sepsis and critical care support in 2002 differed a lot from those nowadays, which could lead to different results. On the other hand, Sakr et al. recruited patients from 24 European countries while Yeo et al.’s study was conducted in multiple centers in Korea. Thus, racial difference may be a possible explanation. We noticed that another prospective single-center study (Chae et al. [15]) from Korea concluded that increased BMIs were not associated with mortality. However, different from Yeo et al.'s study, Chae et al. recruited patients with severe sepsis or septic shock (Yeo et al. recruited patients with sepsis). The association of BMI with mortality was subject to severity of sepsis, which could be a reason for the conflicting results of these two Korean studies.

In addition, there remain some limitations, and issues that need be addressed in future researches. First, whether it is reasonable that BMI is used as the only measure of obesity? Although it is calculated according to weight and height measured in ICU settings, biases are still inevitable, especially considering that patients suspected of sepsis may have received fluid resuscitation treatment before ICU admission. Thus, better measure of adiposity such as BMI combined with waist-to-hip ratio, computed tomography/magnetic resonance or bioelectrical impedance should be considered in future studies. Second, the mechanisms behind effects of obesity on mortality have not been fully understood. As summarized in previous systematic review, increased energy stores, beneficial immunoregulation, inactivation of harmful bacterial products and improved renin–angiotensin–aldosterone system function could play roles [8]. However, for the moment, these are not enough to explain the contradictory results of prospective studies. Third, frailty, which affects both weight and mortality, was not considered in most of included studies. It should be regarded as a confounder and be adjusted for in future studies. Finally, we found significant publication bias, which has never been evaluated in previous meta-analyses. This suggested that studies reporting positive results are easier to be published which may overestimate the effects of increased BMIs.

## Conclusions

Our study indicated that overweight and obese BMIs (25.0–39.9 kg/m^2^) are associated with reduced mortality of patients with sepsis or septic shock, although such survival advantage was not found in all crowds (e.g., patients ≤ 50 years). Considering the protective association of BMI with clinical outcomes found in multiple critical diseases, it seems illogical to negate the discovery of the present meta-analysis. However, the possible BMI measurement bias should not be neglected, and inconsistent results of prospective studies need more reasonable explanations as well. Whether specific factors or potential biases influence the association of BMI with sepsis should be further clarified.

## Supplementary Information

**Additional file 1.** MOOSE checklist for meta-analyses of observational studies.

**Additional file 2.** Search strategies for all databases.

**Additional file 3.** Newcastle-Ottawa Quality Assessment Scale.

**Additional file 4.** The Newcastle-Ottawa Quality Assessment Scale of Included Case-Control or Cohort Studies.

**Additional file 5.** Supplementary figures.

## Data Availability

All data generated or analyzed during this study are included in this published article [14–28].

## Abbreviations

BMI: Body mass index
CI: Confidence interval
HR: Hazard ratio
ICU: Intensive care units
*I*^2^: I-squared
NOS: The Newcastle–Ottawa Scale
OR: Odds ratio
MOOSE: Meta-analysis of Observational Studies in Epidemiology
RR: Relative risk
