# Supplementary material for: Association of body mass index with mortality of sepsis or septic shock: an updated meta-analysis
Source: J Intensive Care. 2023 Jul 3;11:27. doi: 10.1186/s40560-023-00677-0 (PMC10316562; doi:10.1186/s40560-023-00677-0)
Supplement: Supplementary file 1 — Additional file 1. MOOSE checklist for meta-analyses of observational studies. [file 40560_2023_677_MOESM1_ESM.docx]

**Supplementary Appendix 1. MOOSE checklist for meta-analyses of observational studies**

| **Item No** | **Recommendation** | **Reported on Page No** |
| --- | --- | --- |
| Reporting of background should include | | |
| 1 | Problem definition | 5 |
| 2 | Hypothesis statement | 5-6 |
| 3 | Description of study outcome(s) | 6 |
| 4 | Type of exposure or intervention used | 6 |
| 5 | Type of study designs used | 6 |
| 6 | Study population | 6 |
| Reporting of search strategy should include | | |
| 7 | Qualifications of searchers (e.g., librarians and investigators) | 6 |
| 8 | Search strategy, including time period included in the synthesis and key words | 6 |
| 9 | Effort to include all available studies, including contact with authors | 6 |
| 10 | Databases and registries searched | 6 |
| 11 | Search software used, name and version, including special features used (e.g., explosion) | 6 |
| 12 | Use of hand searching (e.g., reference lists of obtained articles) | 6 |
| 13 | List of citations located and those excluded, including justification | 7 |
| 14 | Method of addressing articles published in languages other than English | NA |
| 15 | Method of handling abstracts and unpublished studies | 7 |
| 16 | Description of any contact with authors | NA |
| Reporting of methods should include | | |
| 17 | Description of relevance or appropriateness of studies assembled for assessing the hypothesis to be tested | 6-7 |
| 18 | Rationale for the selection and coding of data (e.g., sound clinical principles or convenience) | 6-7 |
| 19 | Documentation of how data were classified and coded (e.g., multiple raters, blinding and interrater reliability) | 6 |
| 20 | Assessment of confounding (e.g., comparability of cases and controls in studies where appropriate) | 7-8 |
| 21 | Assessment of study quality, including blinding of quality assessors, stratification or regression on possible predictors of study results | 7-8 |
| 22 | Assessment of heterogeneity | 8 |
| 23 | Description of statistical methods (e.g., complete description of fixed or random effects models, justification of whether the chosen models account for predictors of study results, dose-response models, or cumulative meta-analysis) in sufficient detail to be replicated | 8 |
| 24 | Provision of appropriate tables and graphics | 18-23 |
| Reporting of results should include | | |
| 25 | Graphic summarizing individual study estimates and overall estimate | 10 |
| 26 | Table giving descriptive information for each study included | 18-22 |
| 27 | Results of sensitivity testing (e.g., subgroup analysis) | 10-12 |
| 28 | Indication of statistical uncertainty of findings | NA |
| 29 | Quantitative assessment of bias (e.g., publication bias) | 12 |
| 30 | Justification for exclusion (e.g., exclusion of non-English language citations) | 6-7 |
| 31 | Assessment of quality of included studies | 9-10 |
| Reporting of conclusions should include | | |
| 32 | Consideration of alternative explanations for observed results | 15 |
| 33 | Generalization of the conclusions (i.e., appropriate for the data presented and within the domain of the literature review) | 15 |
| 34 | Guidelines for future research | 15 |
| 35 | Disclosure of funding source | 17 |

NA, not applicable.

**eAppendix 2. Search strategies for all databases**

1. PubMed

2. Embase

3. Cochrane Library

4. Web of Science

**1. PubMed**

(("obesity"[Title/Abstract] OR "obese"[Title/Abstract] OR "overweight"[Title/Abstract] OR "body mass index"[Title/Abstract] OR "BMI"[Title/Abstract]) AND ("sepsis"[Title/Abstract] OR "septic"[Title/Abstract]))

**2. Embase**

(obese:ab,ti OR obesity:ab,ti OR overweight:ab,ti OR 'body mass index':ab,ti OR bmi:ab,ti) AND (sepsis:ab,ti OR 'septic shock':ab,ti)

**3. Cochrane Library**

#1 (BMI):ti,ab,kw OR (body mass index):ti,ab,kw OR (obese):ti,ab,kw OR (obesity):ti,ab,kw OR (overweight):ti,ab,kw

#2 (sepsis):ti,ab,kw OR (septic shock):ti,ab,kw

#3 #1 AND #2

**4. Web of Science**

((((((((((TI=(obese)) OR TI=(obesity)) OR TI=(overweight)) OR TI=(BMI)) OR TI=(body mass index)) OR AB=(obese)) OR AB=(obesity)) OR AB=(overweight)) OR AB=(BMI)) OR AB=(body mass index)) AND ((((TI=(septic shock) OR TI=(sepsis)) OR AB=(sepsis)) OR AB=(septic shock)) OR AB=(sepsis))

**eAppendix 3. Newcastle-Ottawa Quality Assessment Scale**

| **Case**-**Control Studies** |
| --- |
| **Selection**  **(1) Is the case definition adequate?**  (a) yes, with independent validation ★  (b) yes, e.g., record linkage or based on self-reports  (c) no description  **(2) Representativeness of the cases**  (a) consecutive or obviously representative series of cases ★  (b) potential for selection biases or not stated  **(3) Selection of Controls**  (a) community controls ★  (b) hospital controls  (c) no description  **(4) Definition of Controls**  (a) no history of disease (endpoint) ★  (b) no description of source |
| **Comparability**  **(1) Comparability of cases and controls on the basis of the design or analysis**  (a) study controls for ____ (Select the most important factor.) ★  (b) study controls for any additional factor (These criteria could be modified to indicate specific control for a second important factor.) ★ |
| **Exposure**  **(1) Ascertainment of exposure**  (a) secure record (e.g., surgical records) ★  (b) structured interview where blind to case/control status ★  (c) interview not blinded to case/control status  (d) written self-report or medical record only  (e) no description  **(2) Same method of ascertainment for cases and controls**  (a) yes ★  (b) no  **(3) Non-Response rate**  (a) same rate for both groups ★  (b) non respondents described  (c) rate different and no designation |
| **Cohort Studies** |
| **Selection**  **(1) Representativeness of the exposed cohort**  (a) truly representative of the average ______ (describe) in the community ★  (b) somewhat representative of the average _____in the community ★  (c) selected group of users e.g., nurses, volunteers  (d) no description of the derivation of the cohort  **(2) Selection of the non-exposed cohort**  (a) drawn from the same community as the exposed cohort ★  (b) drawn from a different source  (c) no description of the derivation of the non-exposed cohort  **(3) Ascertainment of exposure**  (a) secure record (e.g., surgical records) ★  (b) structured interview ★  (c) written self-report  (d) no description  **(4) Demonstration that outcome of interest was not present at start of study**  (a) yes ★  (b) no |
| **Comparability**  **(1) Comparability of cohorts on the basis of the design or analysis**  (a) study controls for ______ (select the most important factor) ★  (b) study controls for any additional factor (These criteria could be modified to indicate specific control for a second important factor.) ★ |
| **Outcome**  **(1) Assessment of outcome**  (a) independent blind assessment ★  (b) record linkage ★  (c) self-report  (d) no description  **(2) Was follow**-**up long enough for outcomes to occur**  (a) yes (select an adequate follow up period for outcome of interest) ★  (b) no  **(3) Adequacy of follow up of cohorts**  (a) complete follow up - all subjects accounted for ★  (b) subjects lost to follow up unlikely to introduce bias - small number lost > ____ % (select an adequate %) follow up, or description provided of those lost) ★  (c) follow up rate < ____% (select an adequate %) and no description of those lost  (d) no statement |

Note: A study can be awarded a maximum of one star for each numbered item within the Selection and Exposure categories. A maximum of two stars can be given for Comparability.

Good quality: 3 or 4 stars in selection domain AND 1 or 2 stars in comparability domain AND 2 or 3 stars in outcome/exposure domain.

Fair quality: 2 stars in selection domain AND 1 or 2 stars in comparability domain AND 2 or 3 stars in outcome/exposure domain.

Poor quality: 0 or 1 star in selection domain OR 0 stars in comparability domain OR 0 or 1 stars in outcome/exposure domain.

**eTable 1. The Newcastle-Ottawa Quality Assessment Scale of Included Case**-**Control or Cohort Studies**

| **Cohort Studies** | | | | | | | | | |
| --- | --- | --- | --- | --- | --- | --- | --- | --- | --- |
| Study | Representativeness of the exposed cohort | Selection of the non-exposed cohort | Ascertainment of exposure | Demonstration that outcome of interest was not present at start of study | Comparability of cohorts on the basis of the design or analysis | Assessment of outcome | Was follow-up long enough for outcomes to occur | Adequacy of follow up of cohorts | Quality Score |
| Wurzinger et al.[1] | ★ | ★ | ★ | ★ | ★★ | ★ | ★ | ★ | 9 |
| Pepper et al.[2] | ★ | ★ | ★ | ★ | ★ | ★ | ★ | 0 | 7 |
| Tay-Lasso et al.[3] | ★ | ★ | ★ | ★ | ★★ | ★ | ★ | 0 | 8 |
| Kuperman et al.[4] | ★ | ★ | ★ | ★ | ★ | ★ | ★ | ★ | 8 |
| Prescott et al.[5] | ★ | ★ | 0 | ★ | ★★ | ★ | ★ | ★ | 8 |
| Sakr et al.[6] | ★ | ★ | 0 | ★ | ★★ | ★ | ★ | ★ | 8 |
| Lin et al.[7] | ★ | ★ | ★ | ★ | ★★ | ★ | ★ | 0 | 8 |
| Danninger et al.[8] | ★ | ★ | ★ | ★ | ★★ | ★ | ★ | ★ | 9 |
| Gaulton et al.[9] | ★ | ★ | ★ | ★ | ★★ | ★ | ★ | ★ | 9 |
| Gaulton et al.[10] | ★ | ★ | ★ | ★ | ★★ | ★ | ★ | ★ | 9 |
| Arabi et al.[11] | ★ | ★ | ★ | ★ | ★★ | ★ | ★ | 0 | 8 |
| Chae et al.[12] | ★ | ★ | ★ | ★ | ★★ | ★ | ★ | ★ | 9 |
| Li et al.[13] | ★ | ★ | ★ | ★ | ★★ | ★ | ★ | 0 | 8 |
| Juarez et al.[14] | ★ | ★ | ★ | ★ | ★ | ★ | ★ | ★ | 8 |
| Yeo et al.[15] | ★ | ★ | ★ | ★ | ★★ | ★ | ★ | ★ | 9 |

eFigure 1A. Individual and pooled results of the association of underweight BMIs with mortality of sepsis in patients > 50 years.


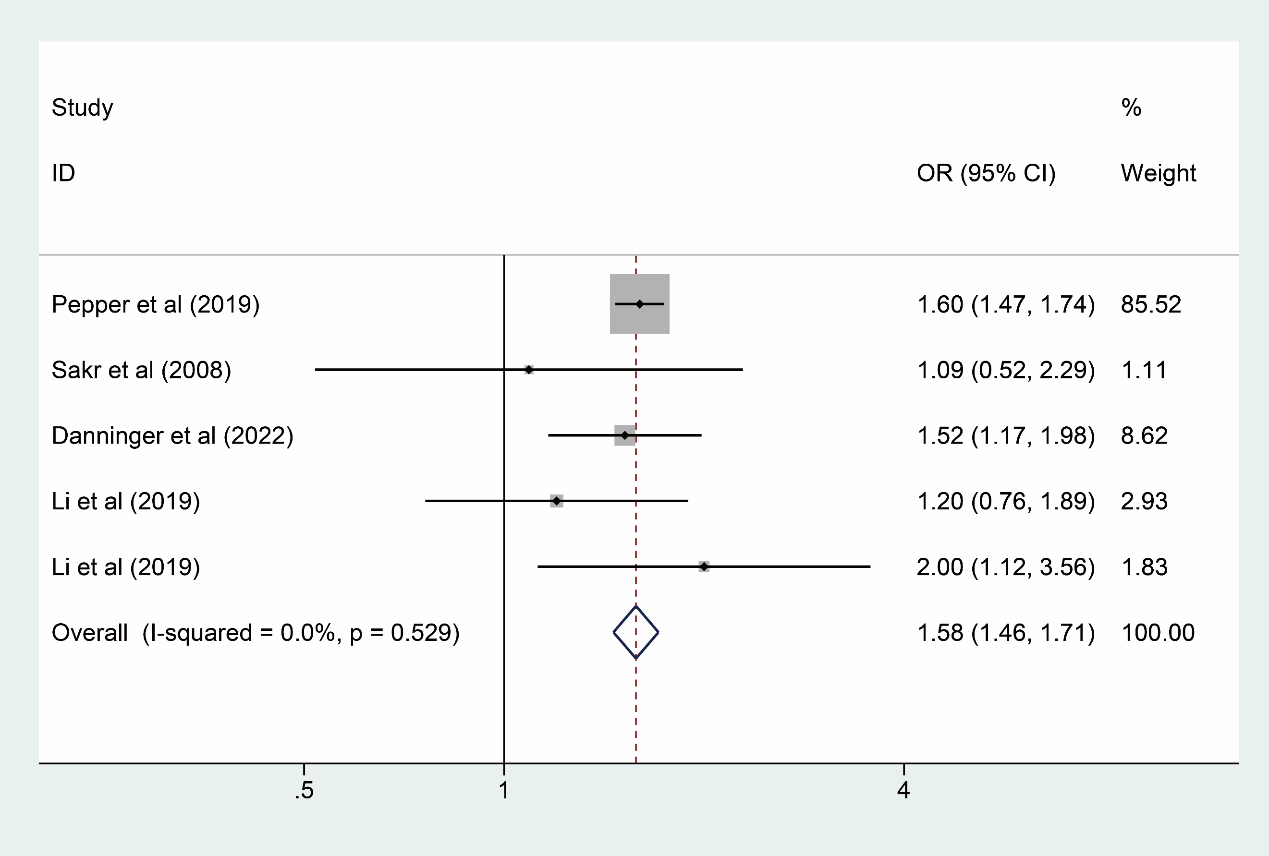


eFigure 1B. Individual and pooled results of the association of underweight BMIs with mortality of sepsis in patients ≤ 50 years.


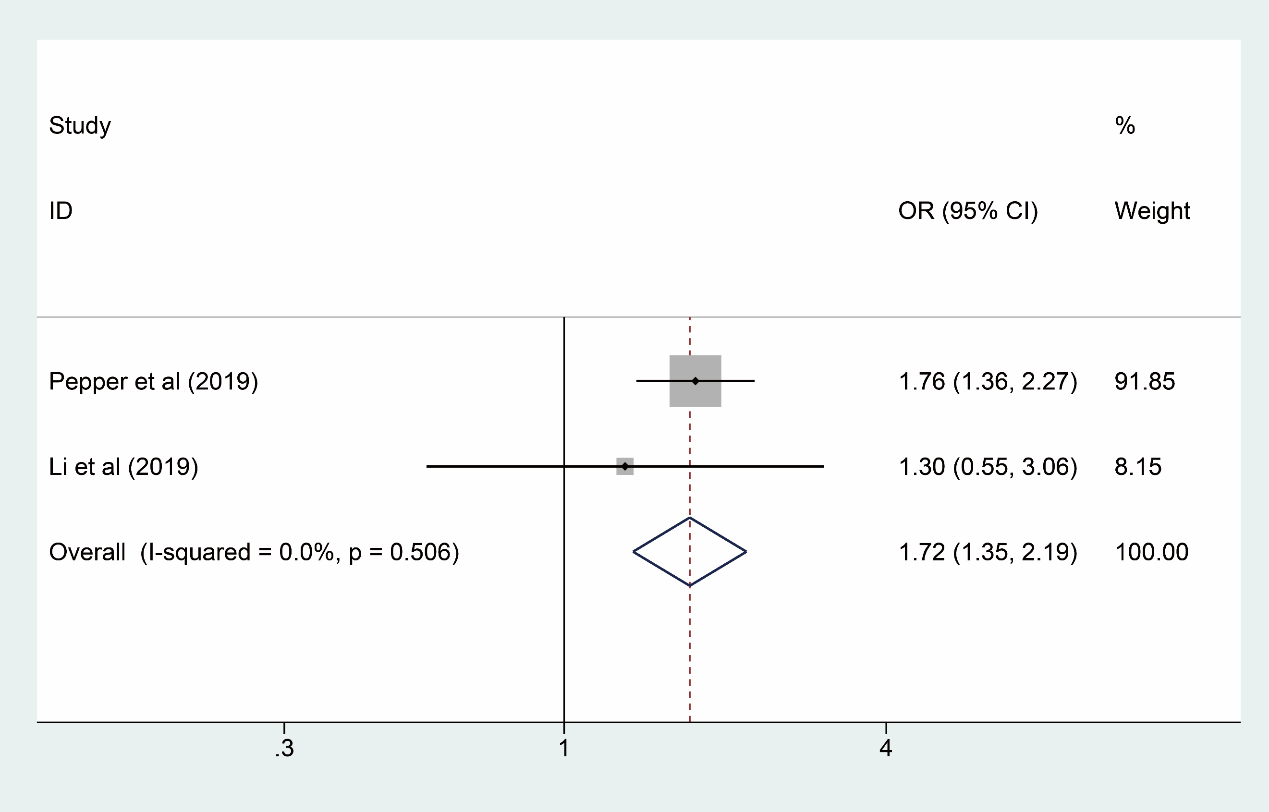


eFigure 1C. Individual and pooled results of the association of overweight BMIs with mortality of sepsis in patients > 50 years.


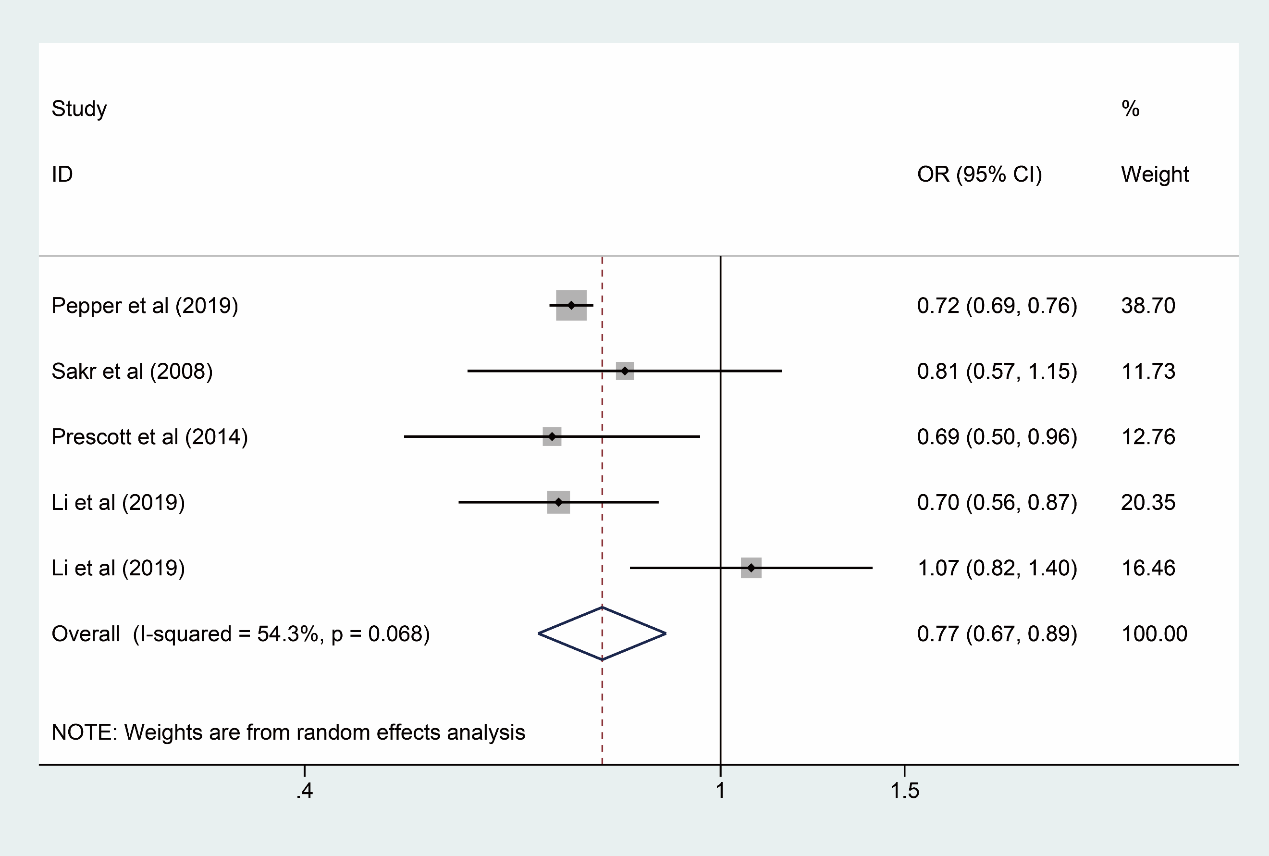


eFigure 1D. Individual and pooled results of the association of overweight BMIs with mortality of sepsis in patients ≤ 50 years.


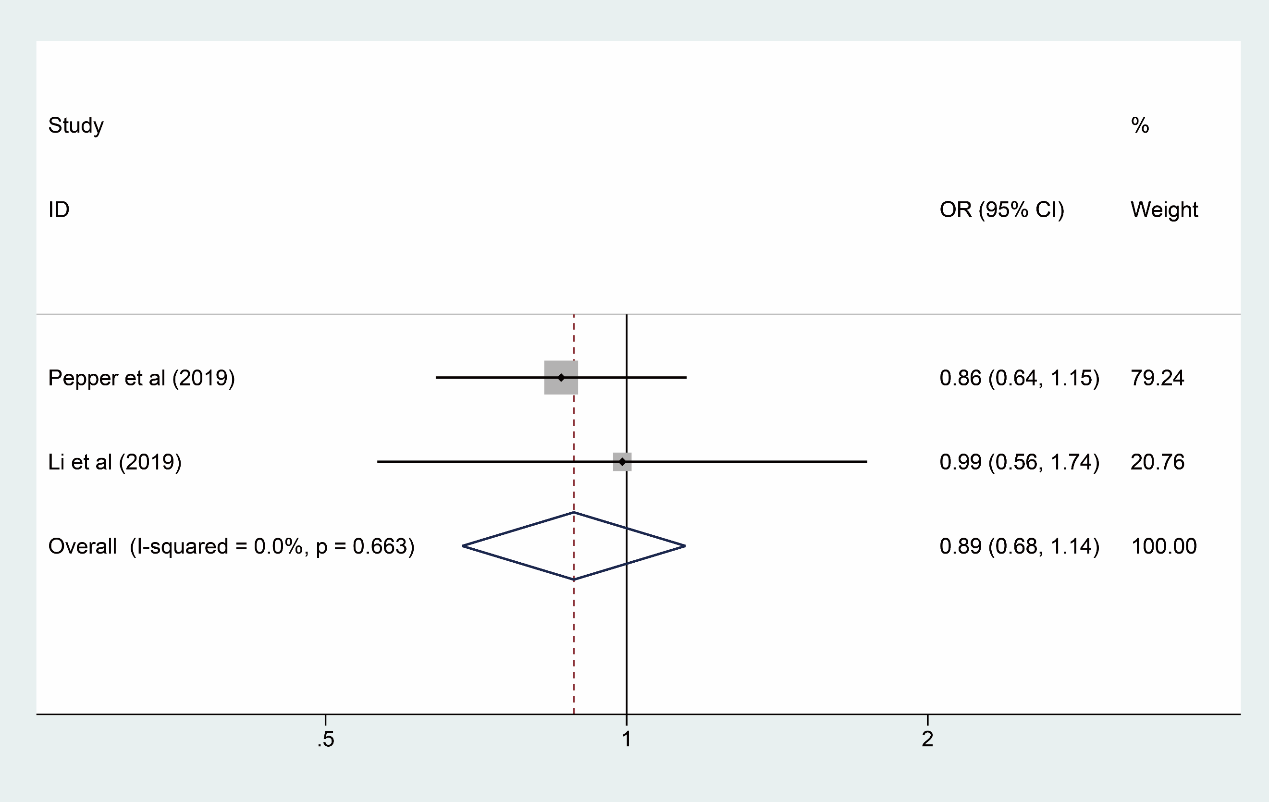


eFigure 1E. Individual and pooled results of the association of obese BMIs with mortality of sepsis in patients > 50 years.


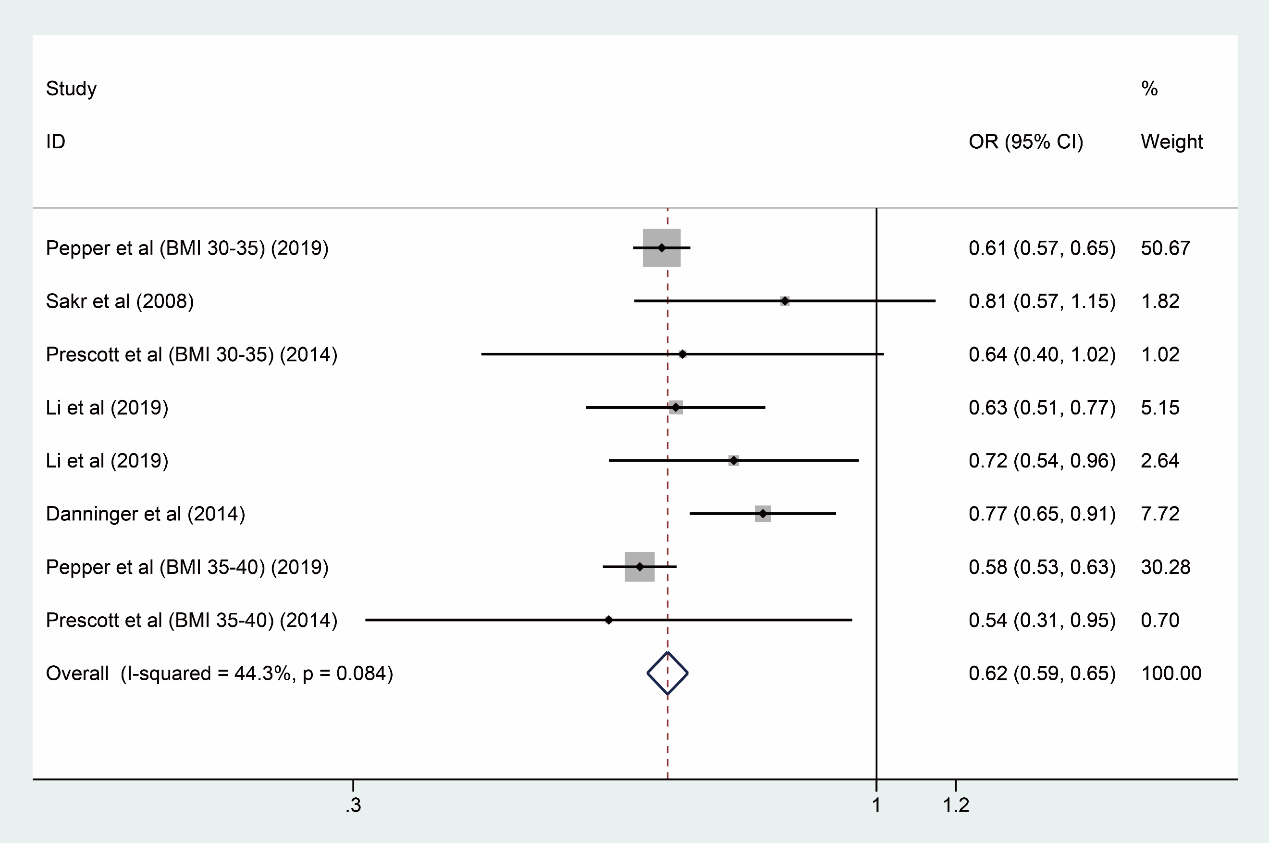


eFigure 1F. Individual and pooled results of the association of obese BMIs with mortality of sepsis in patients ≤ 50 years.


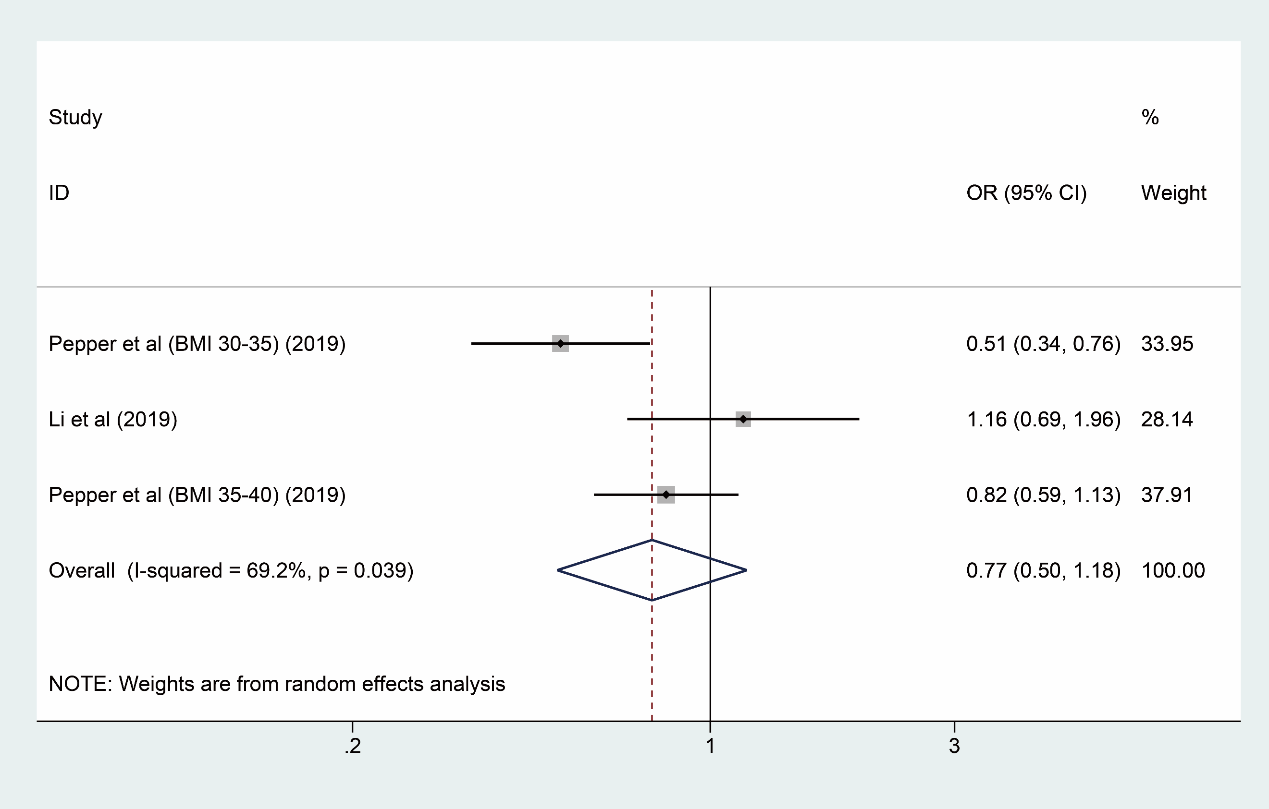


eFigure 2A. Individual and pooled results of the association of underweight BMIs with mortality of sepsis in retrospective studies.


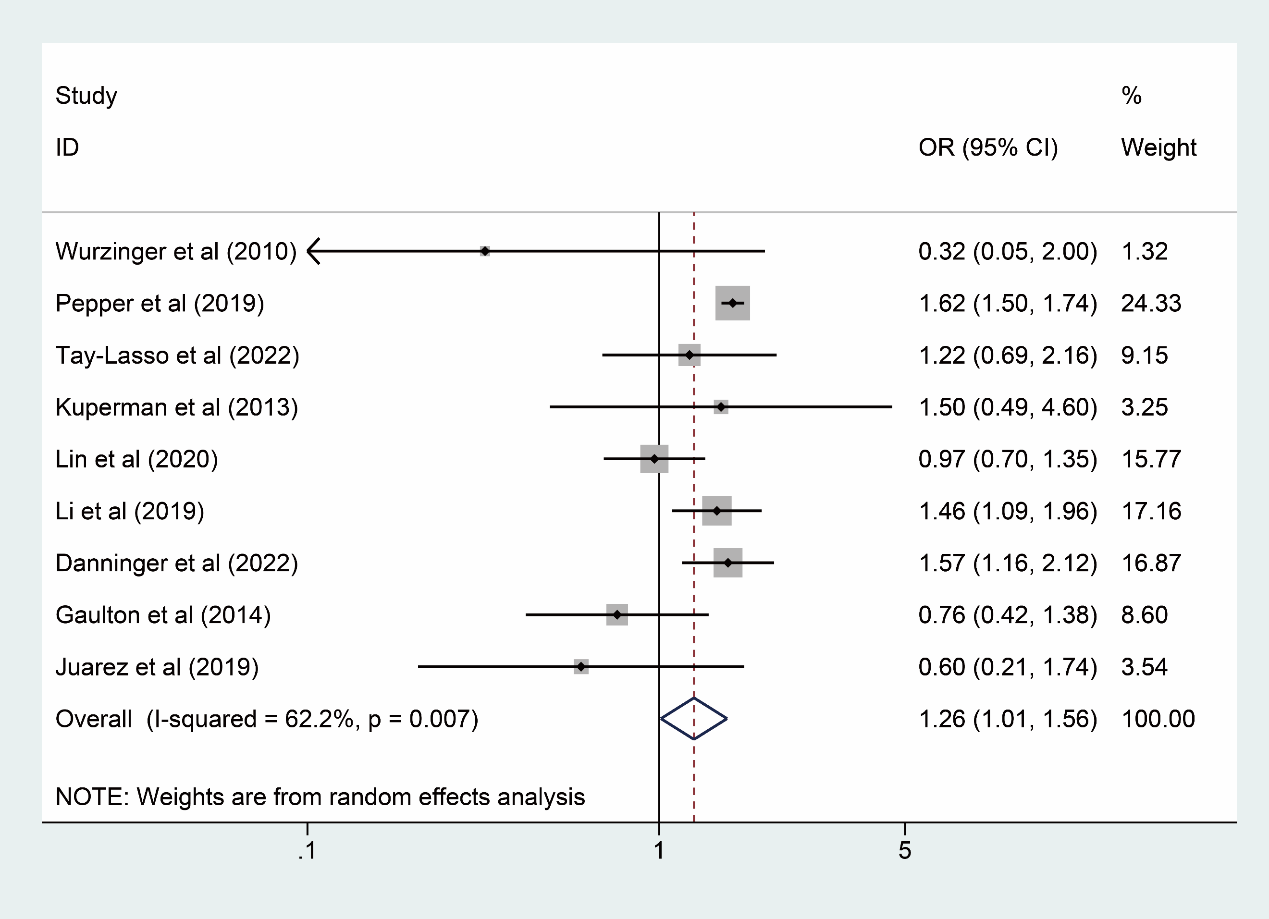


eFigure 2B. Individual and pooled results of the association of underweight BMIs with mortality of sepsis in prospective studies.


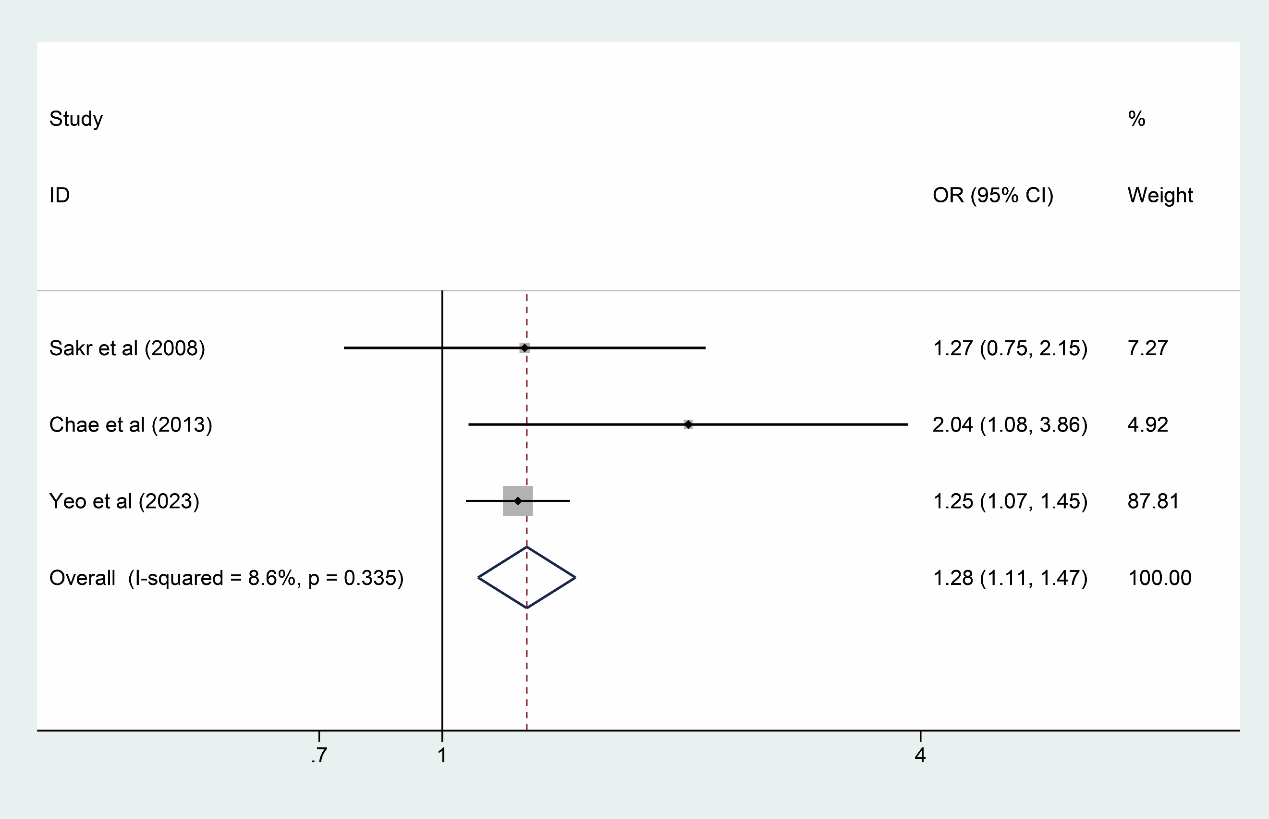


eFigure 2C. Individual and pooled results of the association of overweight BMIs with mortality of sepsis in retrospective studies.


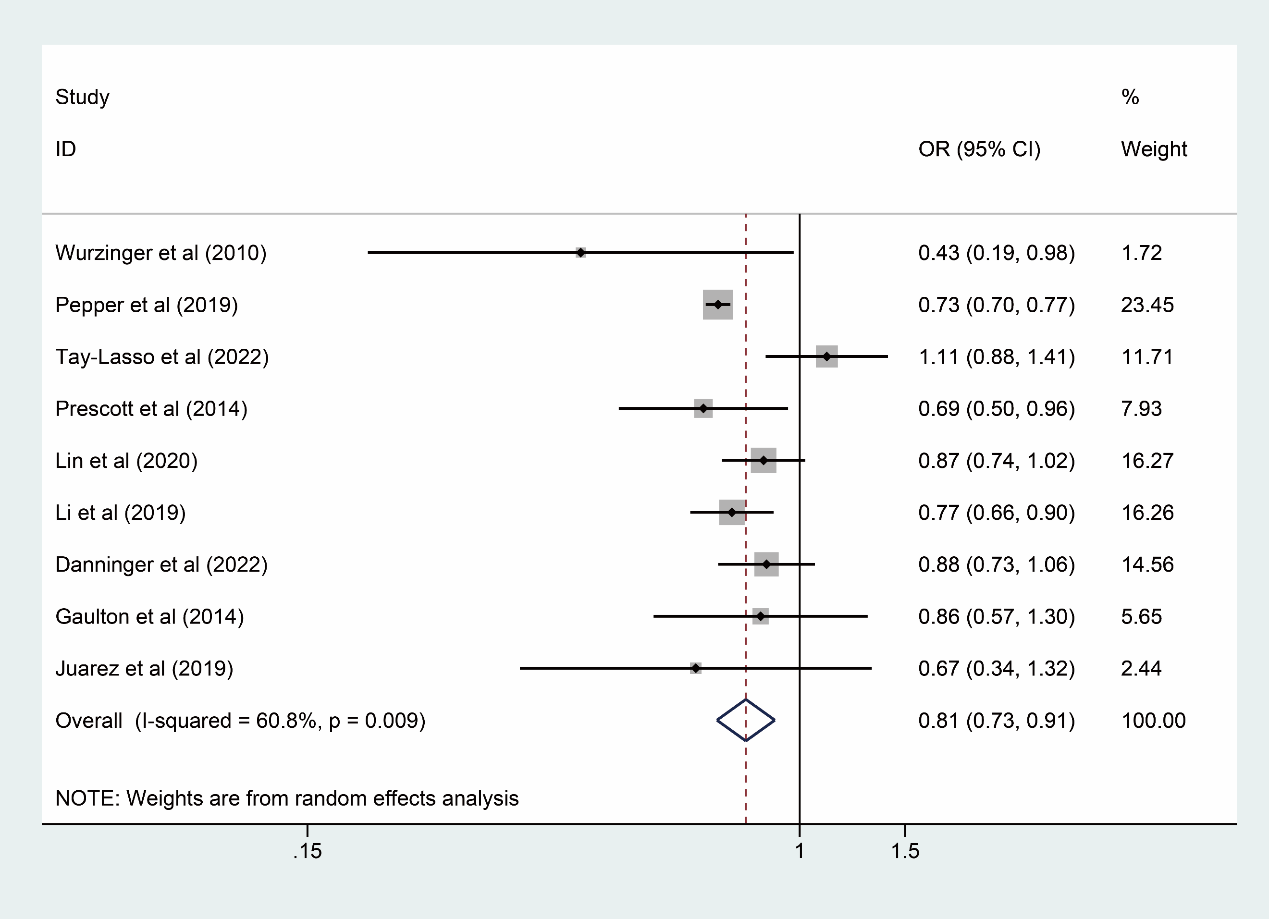


eFigure 2D. Individual and pooled results of the association of overweight BMIs with mortality of sepsis in prospective studies.


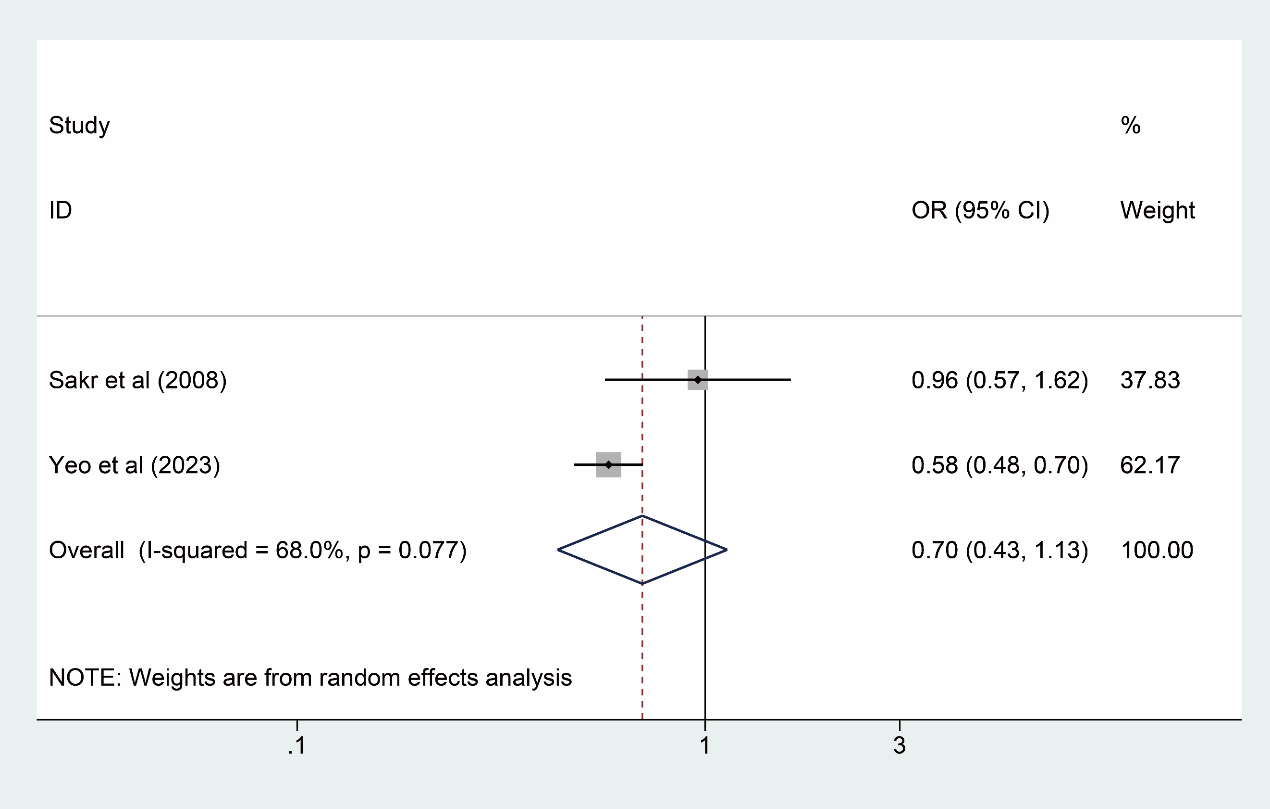


eFigure 2E. Individual and pooled results of the association of obese BMIs with mortality of sepsis in retrospective studies.


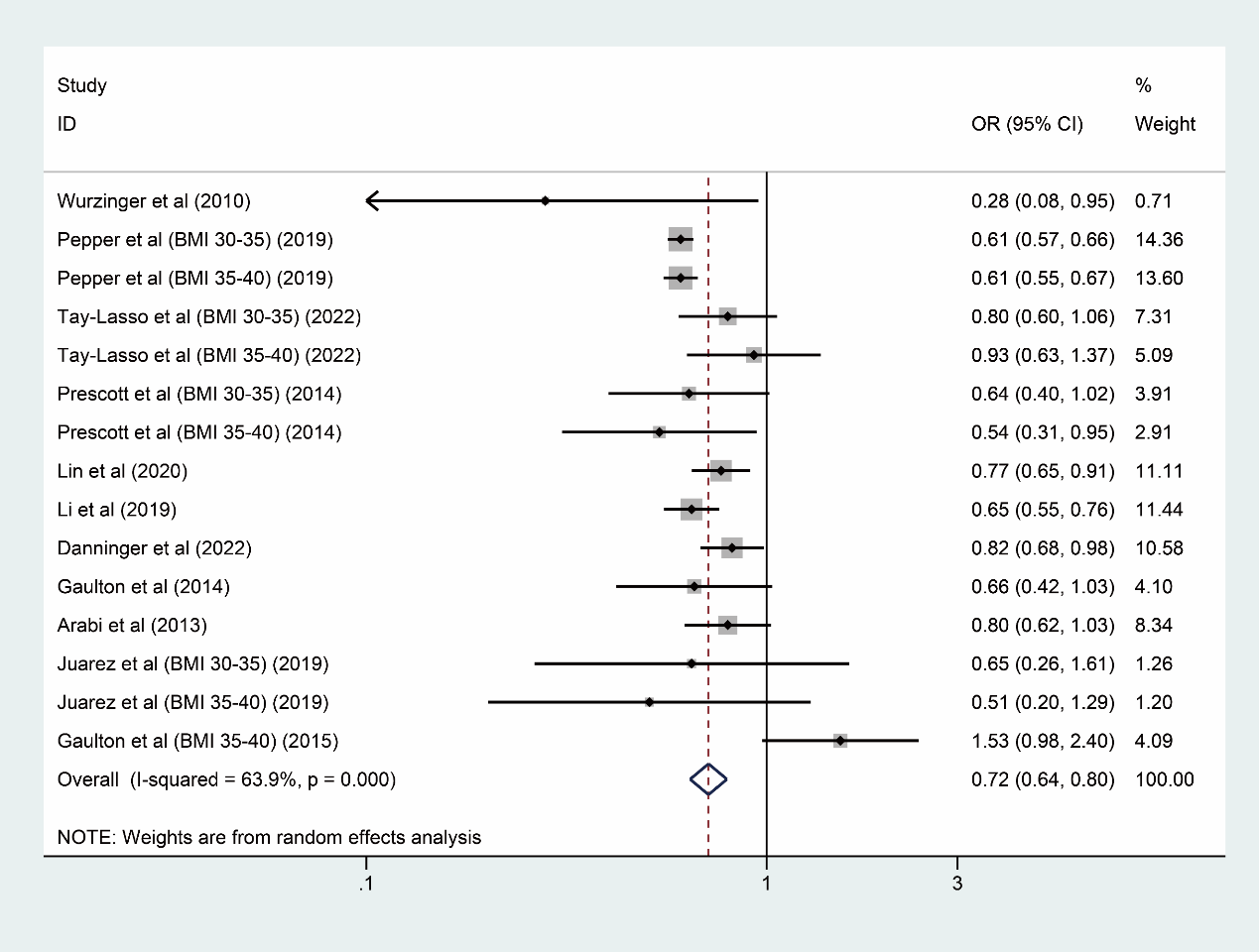


eFigure 2F. Individual and pooled results of the association of obese BMIs with mortality of sepsis in prospective studies.


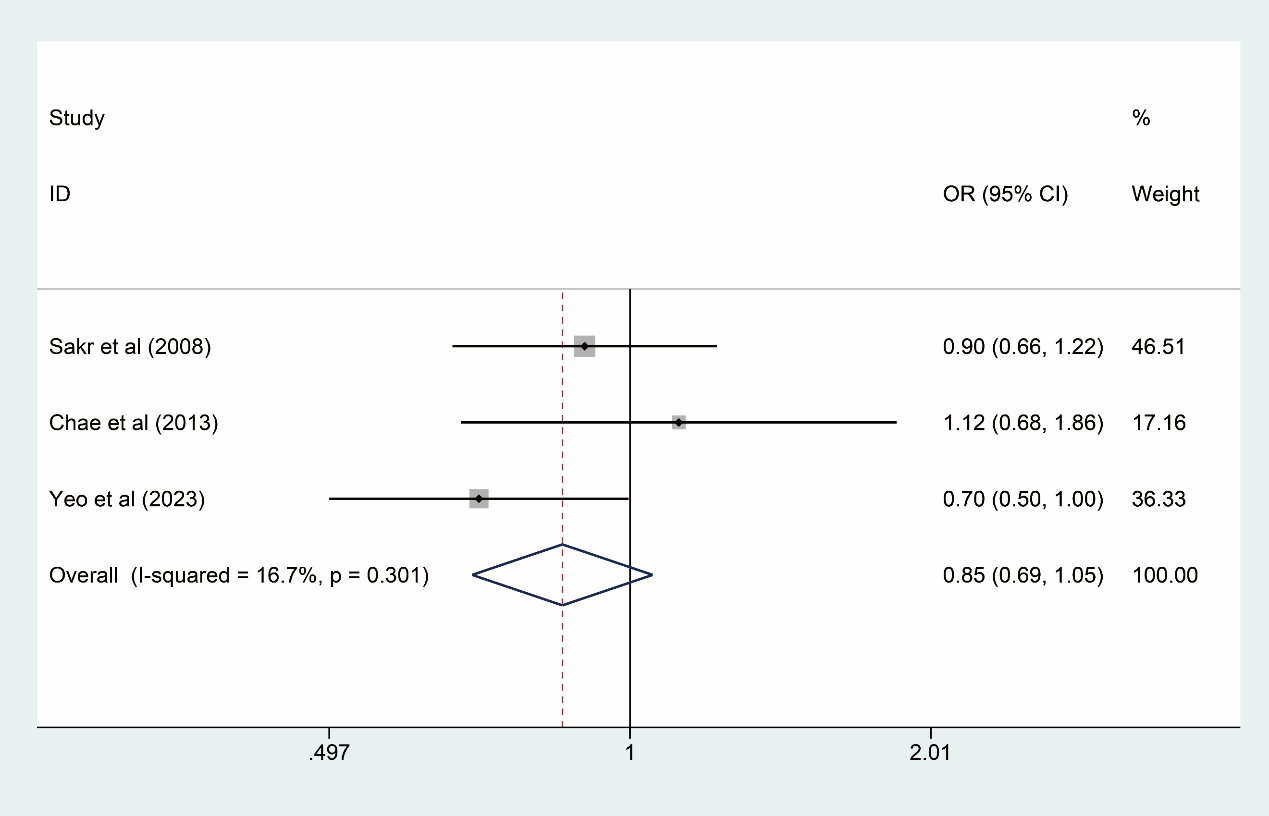


eFigure 3A. Individual and pooled results of the association of underweight BMIs with mortality of sepsis in studies where diagnoses were based on Sepsis 1.0 or 2.0 criteria.


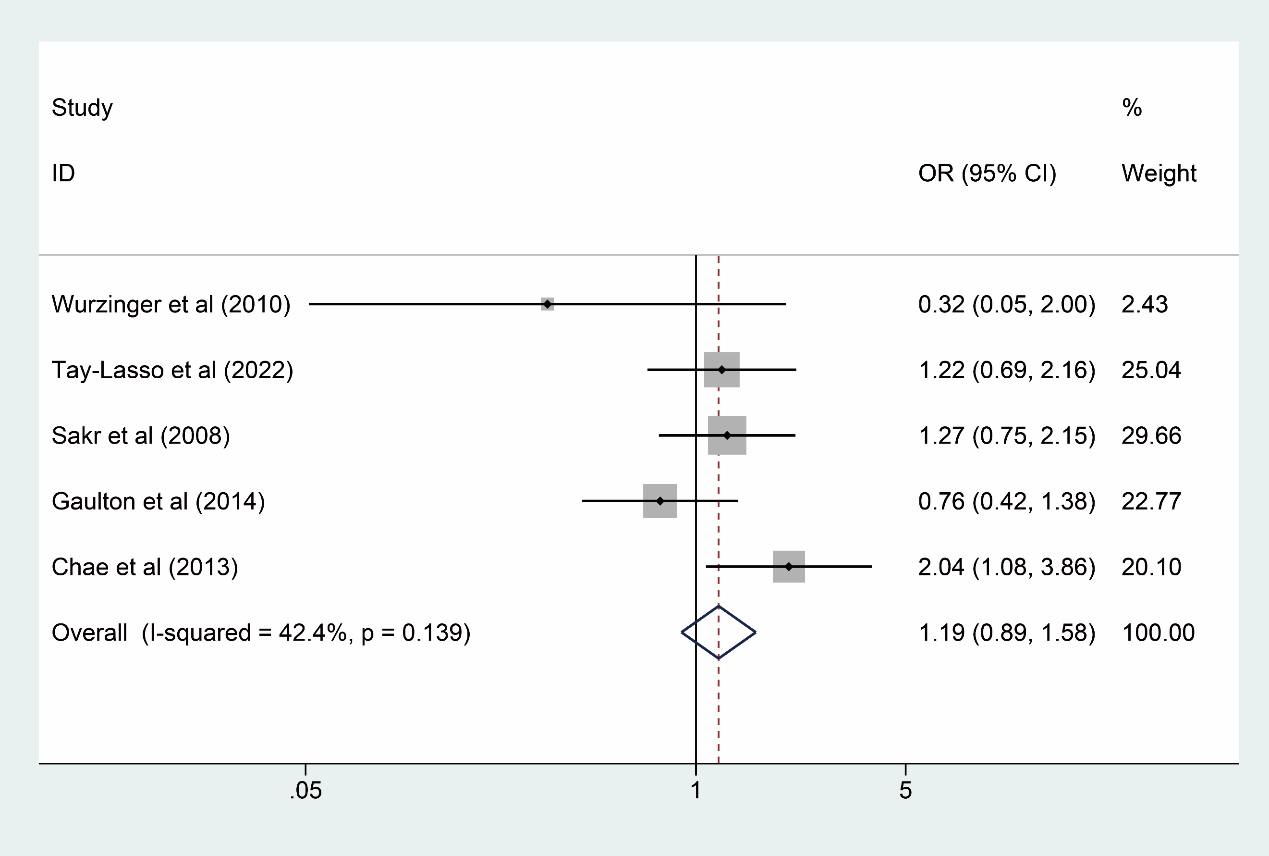


eFigure 3B. Individual and pooled results of the association of underweight BMIs with mortality of sepsis in studies where diagnoses were based on Sepsis 3.0 criteria.


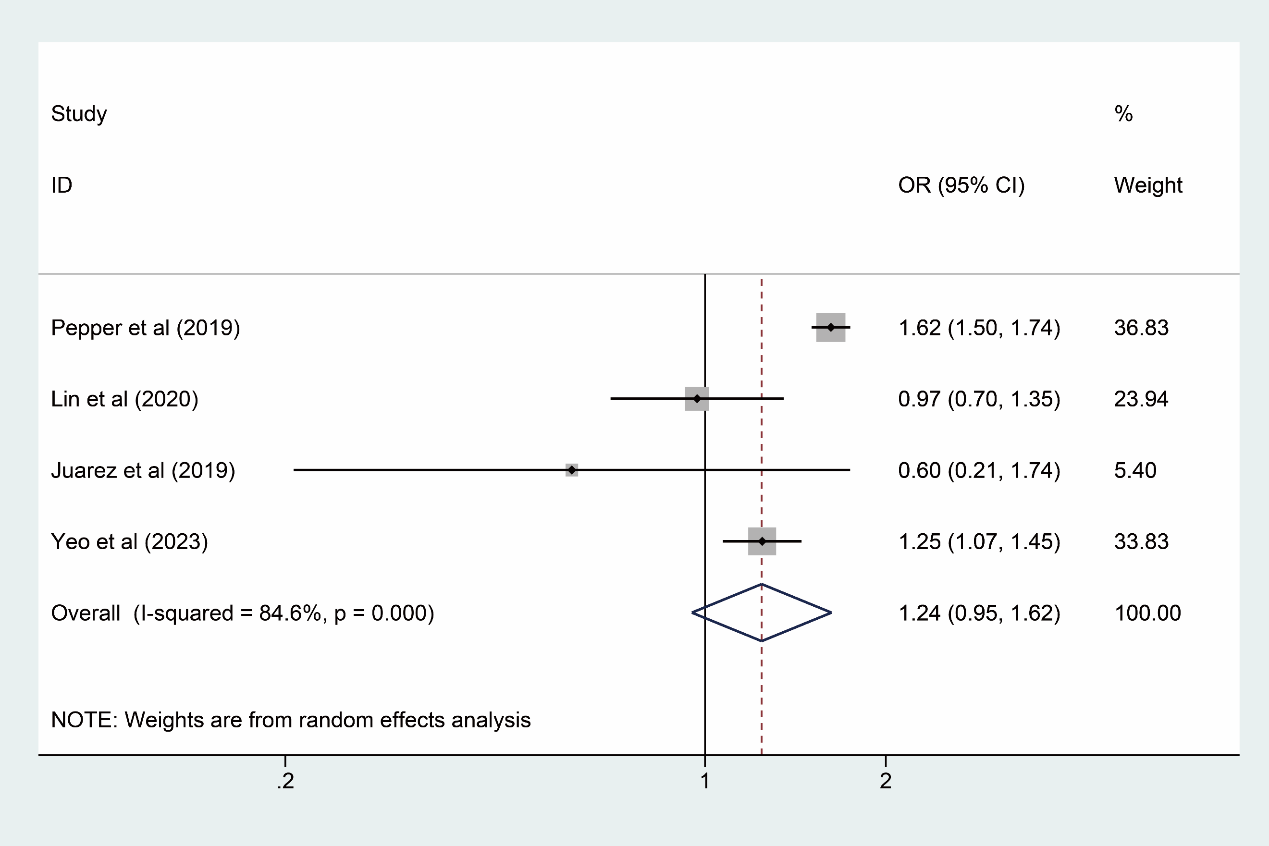


eFigure 3C. Individual and pooled results of the association of overweight BMIs with mortality of sepsis in studies where diagnoses were based on Sepsis 1.0 or 2.0 criteria.


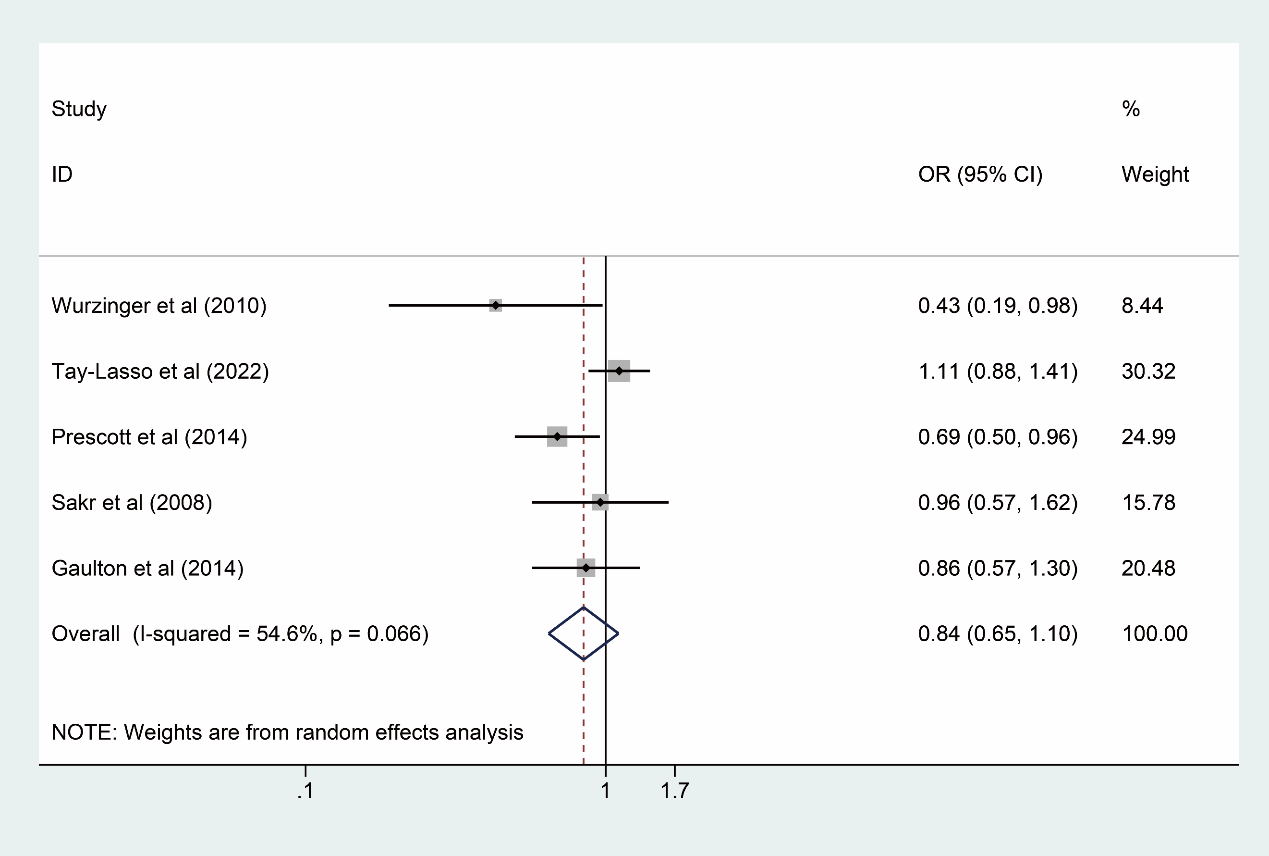


eFigure 3D. Individual and pooled results of the association of overweight BMIs with mortality of sepsis in studies where diagnoses were based on Sepsis 3.0 criteria.


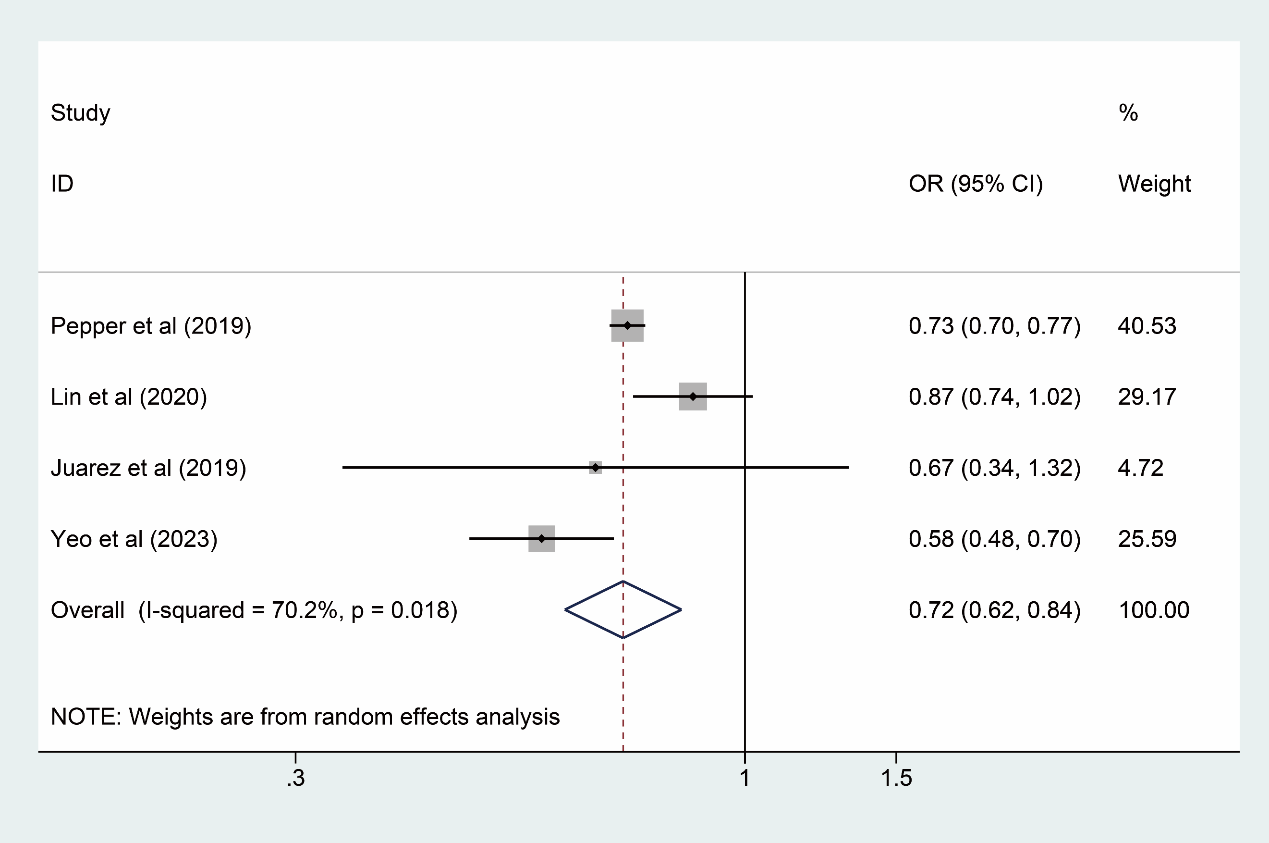


eFigure 3E. Individual and pooled results of the association of obese BMIs with mortality of sepsis in studies where diagnoses were based on Sepsis 1.0 or 2.0 criteria.


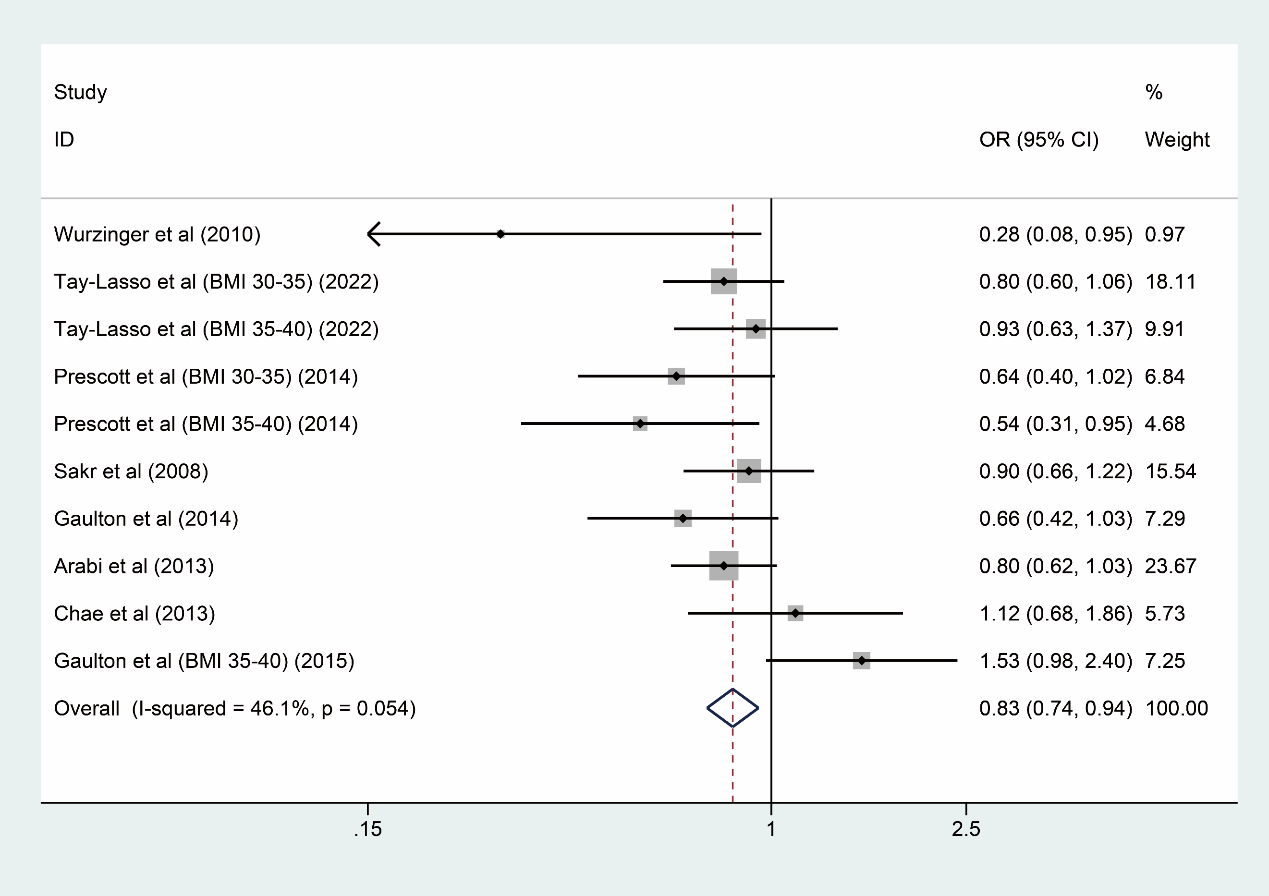


eFigure 3F. Individual and pooled results of the association of obese BMIs with mortality of sepsis in studies where diagnoses were based on Sepsis 3.0 criteria.


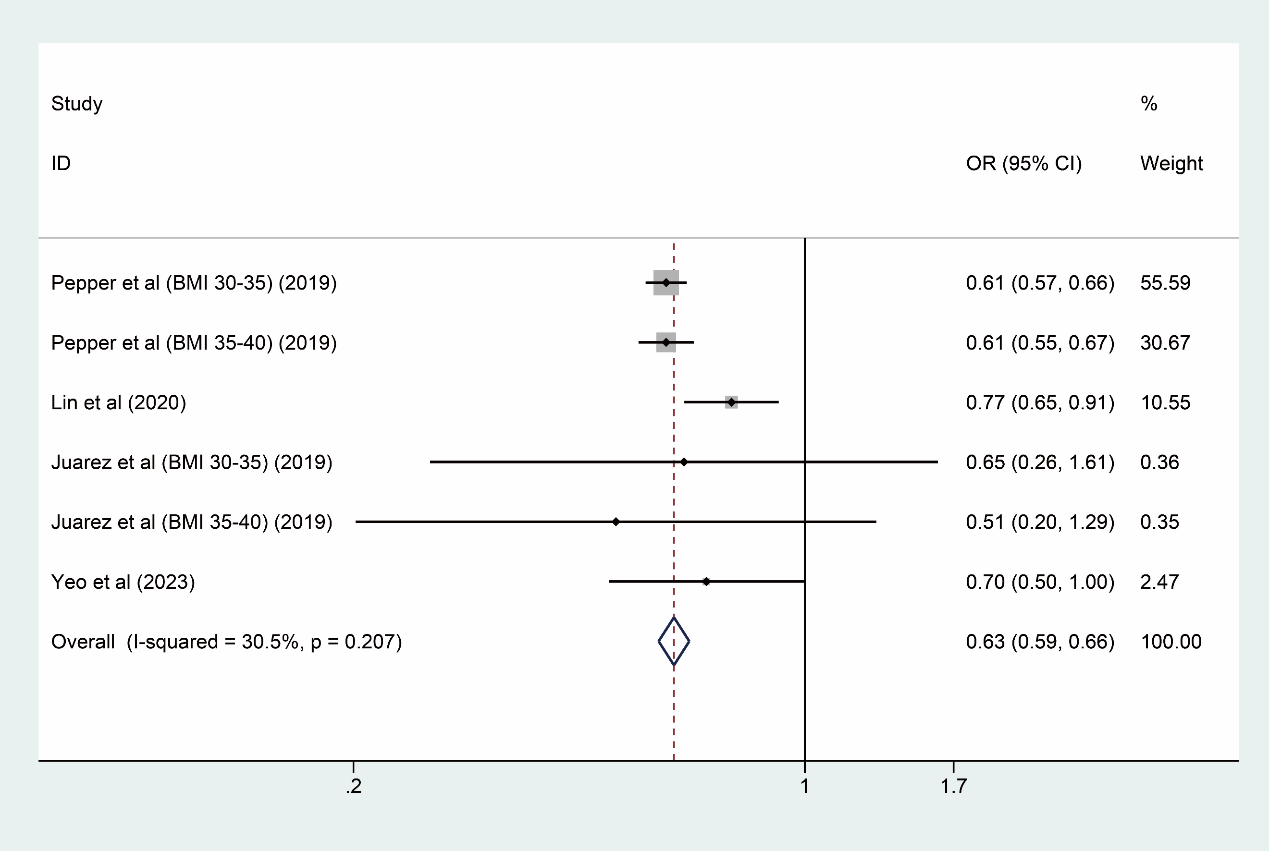


eFigure 4A. Individual and pooled results of the association of underweight BMIs with mortality in patients diagnosed with sepsis.


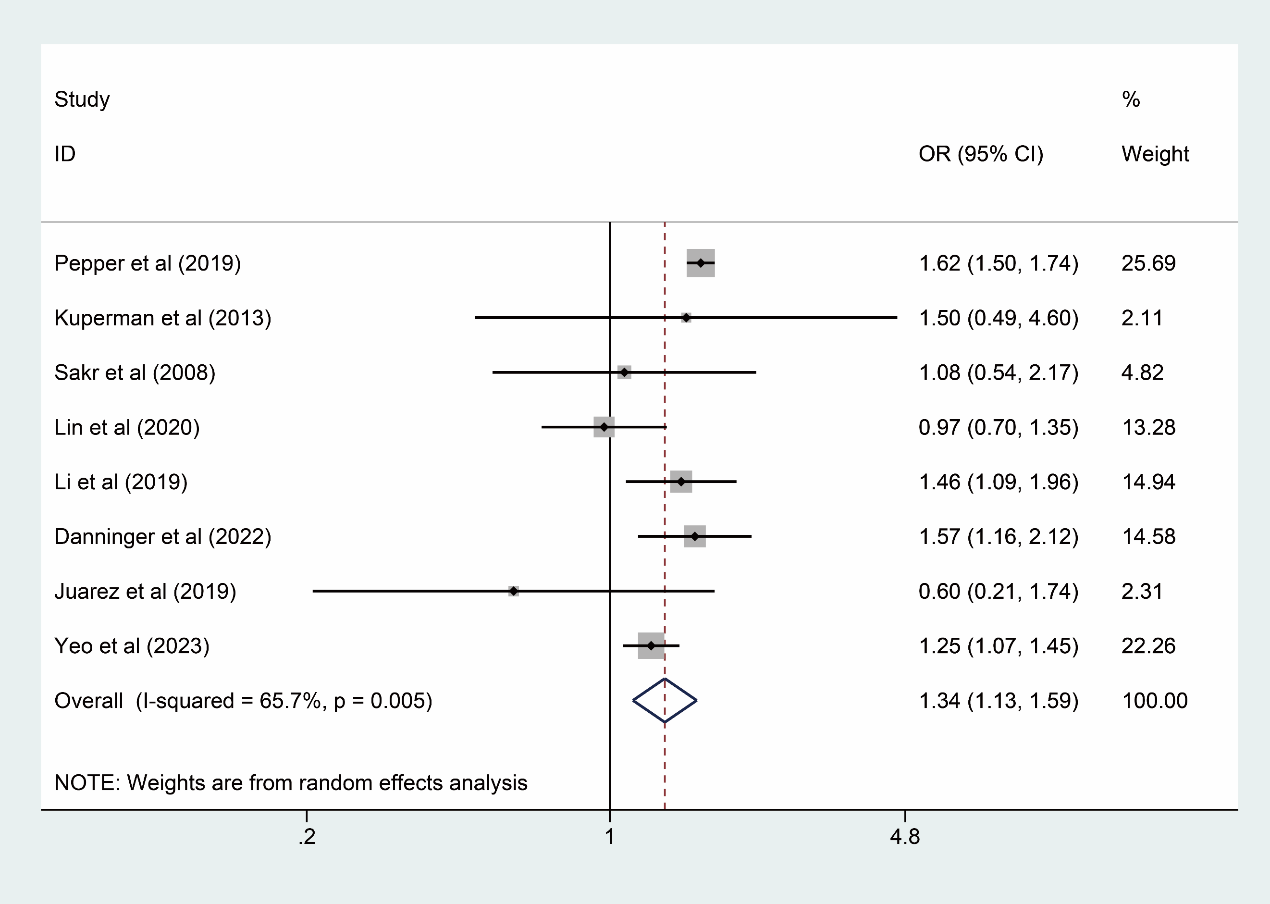


eFigure 4B. Individual and pooled results of the association of underweight BMIs with mortality in patients diagnosed with severe sepsis or septic shock.


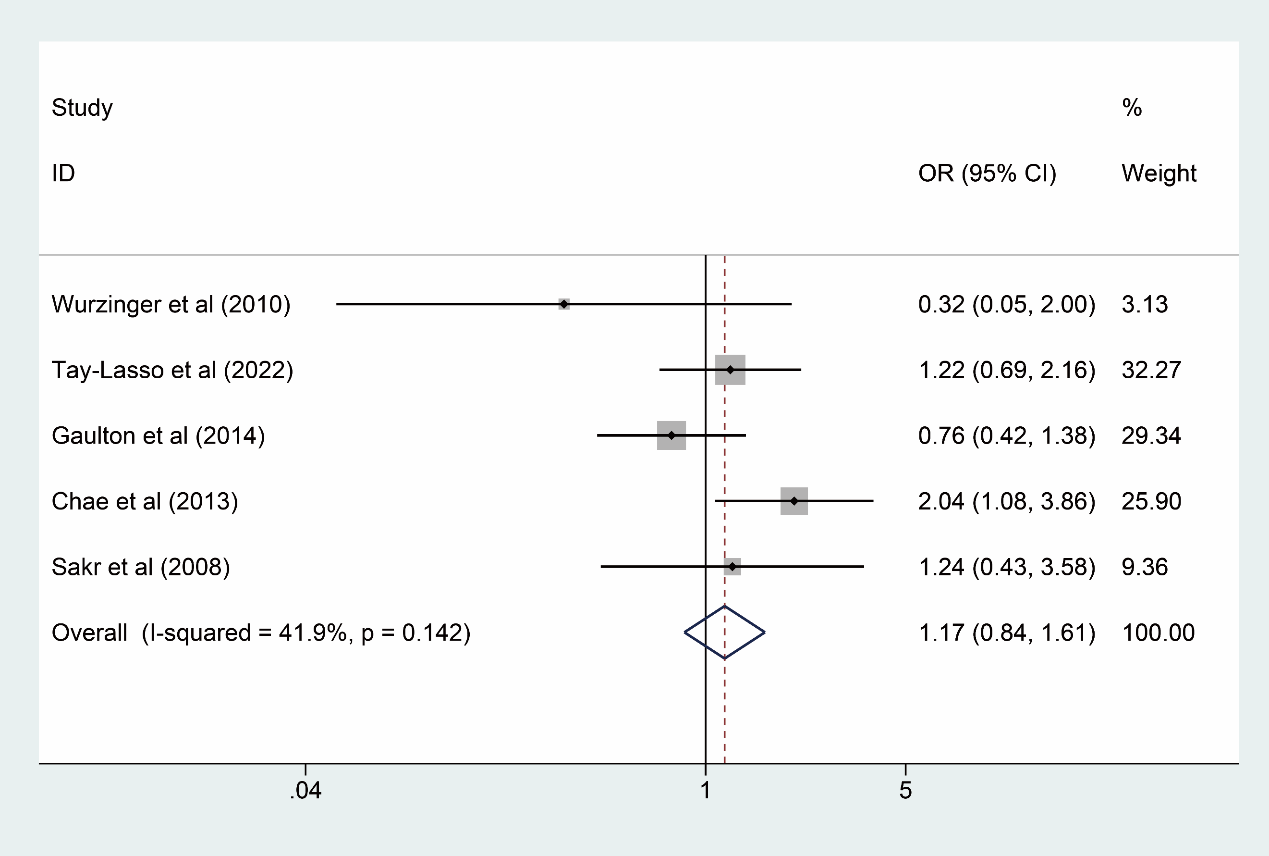


eFigure 4C. Individual and pooled results of the association of overweight BMIs with mortality in patients diagnosed with sepsis.


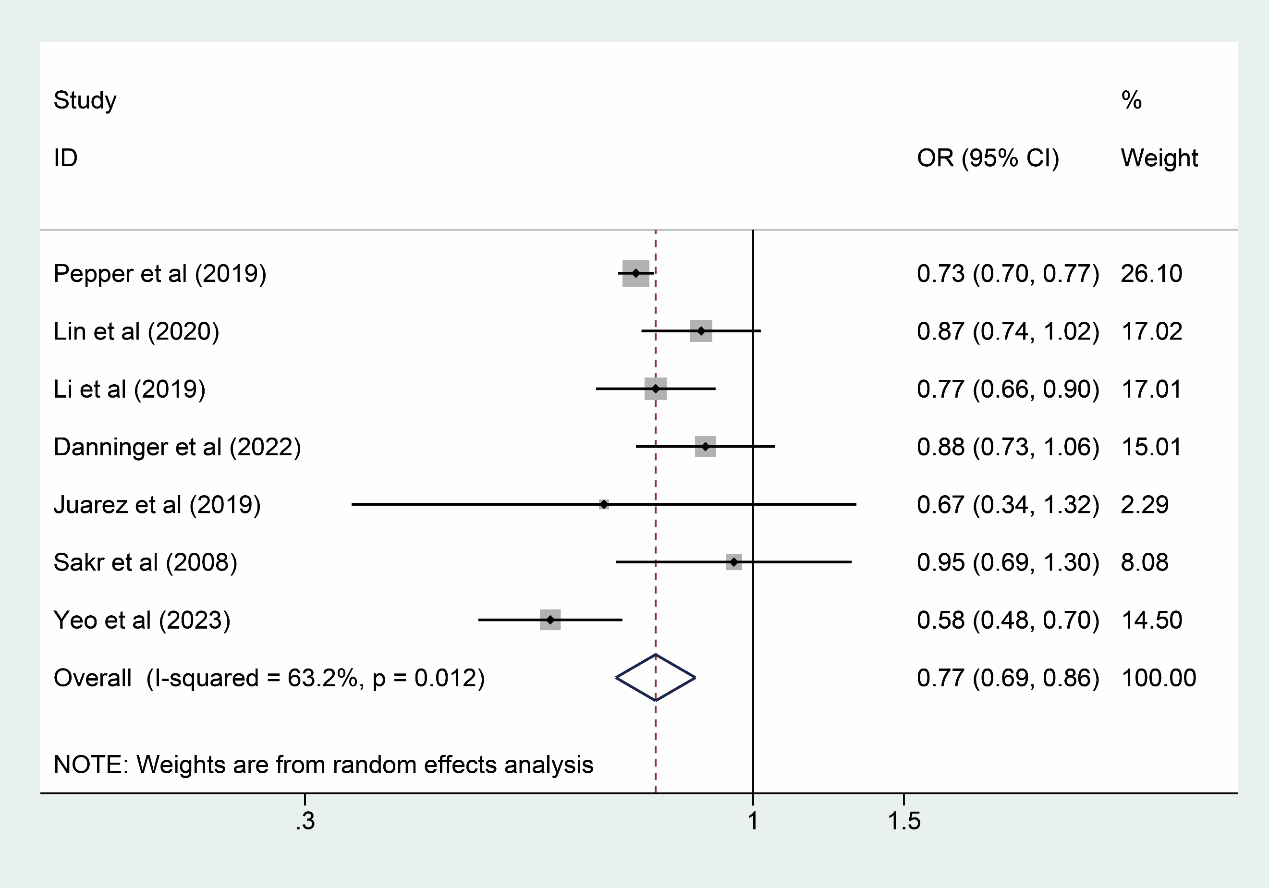


eFigure 4D. Individual and pooled results of the association of overweight BMIs with mortality in patients diagnosed with severe sepsis or septic shock.


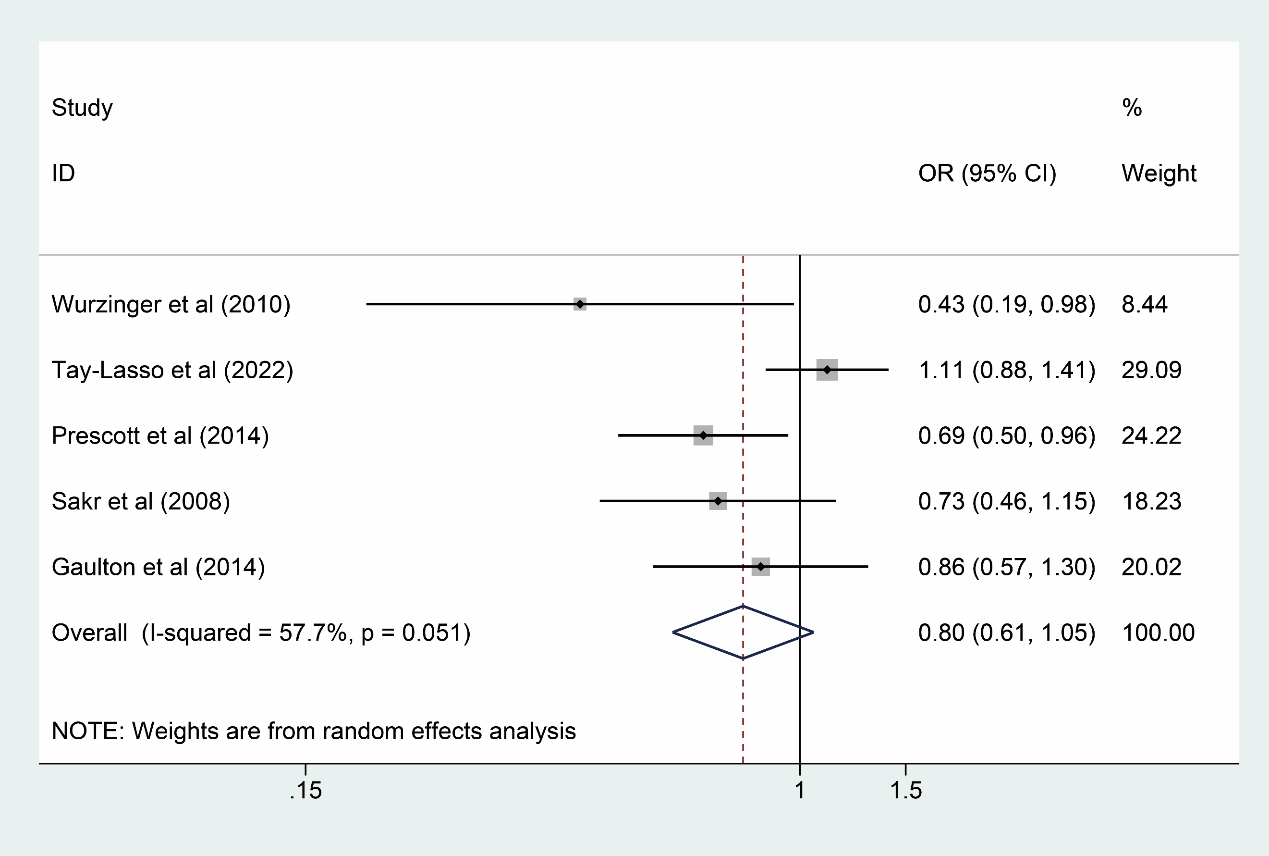


eFigure 4E. Individual and pooled results of the association of obese BMIs with mortality in patients diagnosed with sepsis.


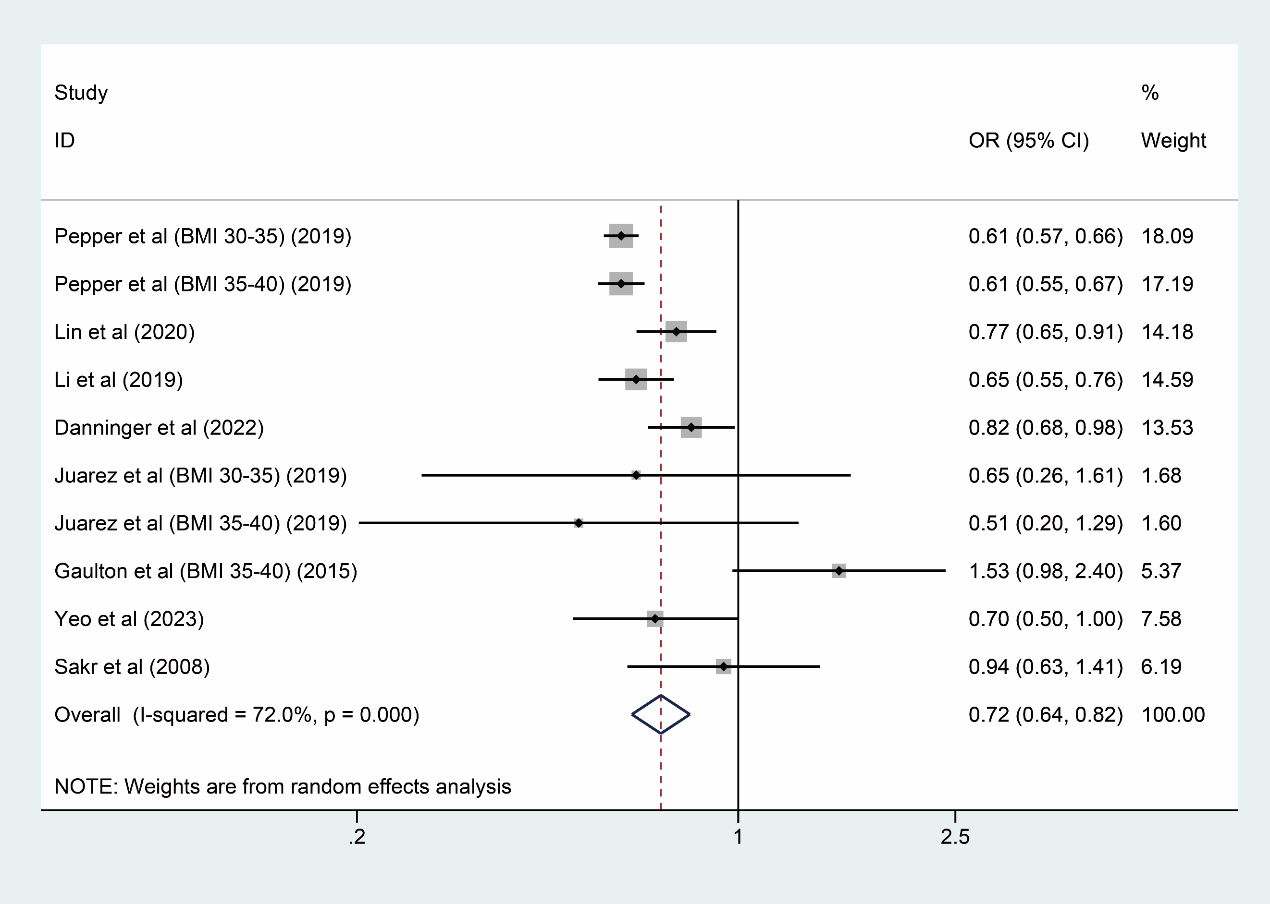


eFigure 4F. Individual and pooled results of the association of obese BMIs with mortality in patients diagnosed with severe sepsis or septic shock.


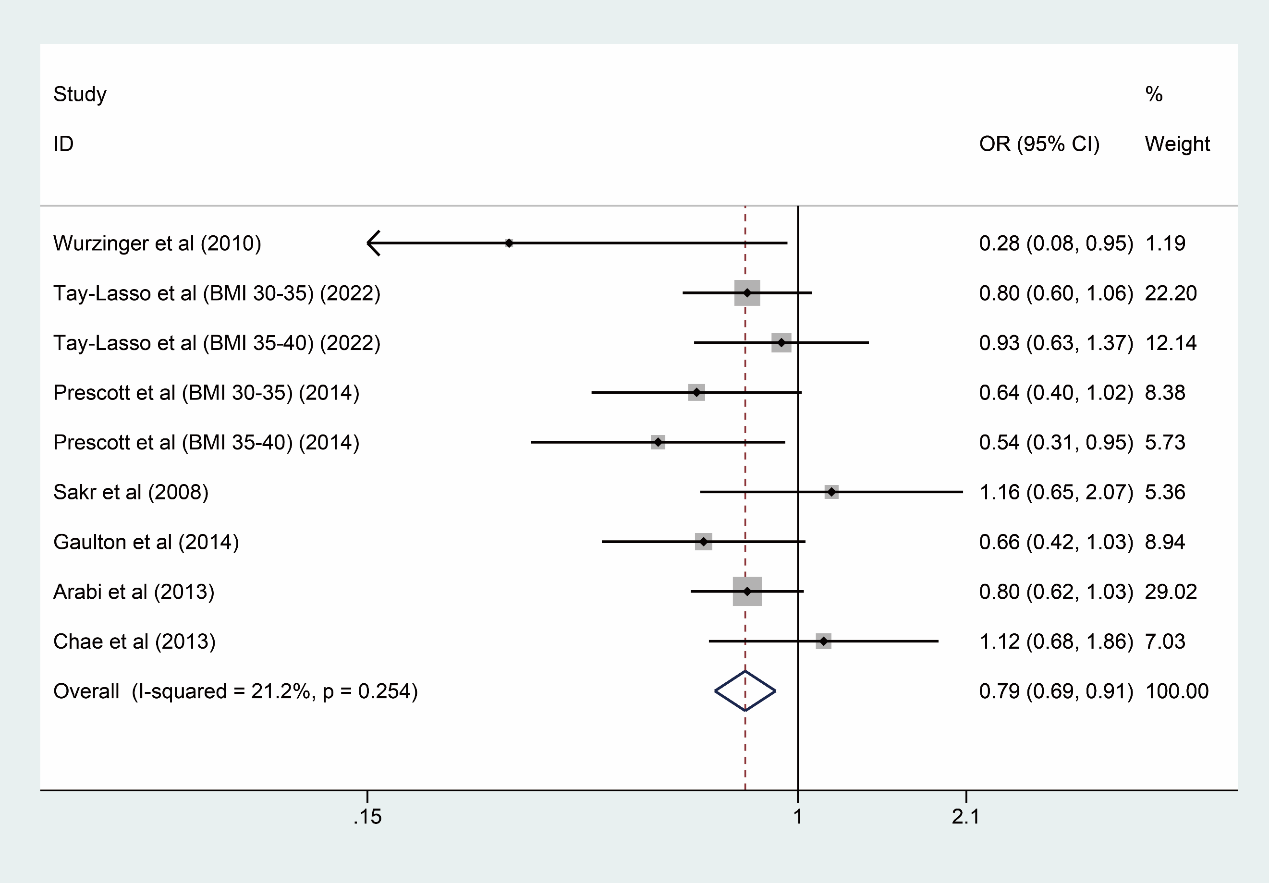


**Reference**

1. Wurzinger B, Dünser MW, Wohlmuth C, Deutinger MC, Ulmer H, Torgersen C, et al. The association between body-mass index and patient outcome in septic shock: a retrospective cohort study. Wiener klinische Wochenschrift. 2010;122(1-2):31-6.

2. Pepper DJ, Demirkale CY, Sun J, Rhee C, Fram D, Eichacker P, et al. Does Obesity Protect Against Death in Sepsis? A Retrospective Cohort Study of 55,038 Adult Patients. Critical care medicine. 2019;47(5):643-50.

3. Tay-Lasso E, Grigorian A, Lekawa M, Dolich M, Schubl S, Barrios C, et al. Obesity Does Not Increase Risk for Mortality in Severe Sepsis Trauma Patients. The American surgeon. 2022:31348221078986.

4. Kuperman EF, Showalter JW, Lehman EB, Leib AE, Kraschnewski JL. The impact of obesity on sepsis mortality: a retrospective review. BMC infectious diseases. 2013;13:377.

5. Prescott HC, Chang VW, O'Brien JM, Jr., Langa KM, Iwashyna TJ. Obesity and 1-year outcomes in older Americans with severe sepsis. Critical care medicine. 2014;42(8):1766-74.

6. Sakr Y, Madl C, Filipescu D, Moreno R, Groeneveld J, Artigas A, et al. Obesity is associated with increased morbidity but not mortality in critically ill patients. Intensive care medicine. 2008;34(11):1999-2009.

7. Lin S, Ge S, He W, Zeng M. Association between Body Mass Index and Short-Term Clinical Outcomes in Critically Ill Patients with Sepsis: A Real-World Study. BioMed research international. 2020;2020:5781913.

8. Danninger T, Rezar R, Mamandipoor B, Dankl D, Koköfer A, Jung C, et al. Underweight but not overweight is associated with excess mortality in septic ICU patients. Wiener klinische Wochenschrift. 2022;134(3-4):139-47.

9. Gaulton TG, Marshall MacNabb C, Mikkelsen ME, Agarwal AK, Cham Sante S, Shah CV, et al. A retrospective cohort study examining the association between body mass index and mortality in severe sepsis. Internal and emergency medicine. 2015;10(4):471-9.

10. Gaulton TG, Weiner MG, Morales KH, Gaieski DF, Mehta J, Lautenbach E. The effect of obesity on clinical outcomes in presumed sepsis: a retrospective cohort study. Internal and emergency medicine. 2014;9(2):213-21.

11. Arabi YM, Dara SI, Tamim HM, Rishu AH, Bouchama A, Khedr MK, et al. Clinical characteristics, sepsis interventions and outcomes in the obese patients with septic shock: an international multicenter cohort study. Critical care (London, England). 2013;17(2):R72.

12. Chae MK CD, Shin TG, Jeon K, Suh GY, Sim MS, Song KJ, Jeong YK, Jo IJ. Body Mass Index and Outcomes in Patients with Severe Sepsis or Septic Shock. Korean Journal of Critical Care Medicine. 2013;28(4):266-71.

13. Li S, Hu X, Xu J, Huang F, Guo Z, Tong L, et al. Increased body mass index linked to greater short- and long-term survival in sepsis patients: A retrospective analysis of a large clinical database. International journal of infectious diseases : IJID : official publication of the International Society for Infectious Diseases. 2019;87:109-16.

14. Juarez E EH, Lear M, Sanchez A, Yang S, Nugent K. The association between body mass index and outcomes in patient with sepsis and acute respiratory failure. The Southwest Respiratory and Critical Care Chronicles. 2019;7(31):13-23.

15. Yeo HJ, Kim TH, Jang JH, Jeon K, Oh DK, Park MH, et al. Obesity Paradox and Functional Outcomes in Sepsis: A Multicenter Prospective Study. Critical care medicine. 2023.
