# Supplementary material for: Association of body mass index with mortality of sepsis or septic shock: an updated meta-analysis
Source: J Intensive Care. 2023 Jul 3;11:27. doi: 10.1186/s40560-023-00677-0 (PMC10316562; doi:10.1186/s40560-023-00677-0)
Supplement: Supplementary file 2 — Additional file 2. Search strategies for all databases. [file 40560_2023_677_MOESM2_ESM.docx]

**Search strategies for all databases**

1. PubMed

2. Embase

3. Cochrane Library

4. Web of Science

**1. PubMed**

(("obesity"[Title/Abstract] OR "obese"[Title/Abstract] OR "overweight"[Title/Abstract] OR "body mass index"[Title/Abstract] OR "BMI"[Title/Abstract]) AND ("sepsis"[Title/Abstract] OR "septic"[Title/Abstract]))

2205 records

**2. Embase**

(obese:ab,ti OR obesity:ab,ti OR overweight:ab,ti OR 'body mass index':ab,ti OR bmi:ab,ti) AND (sepsis:ab,ti OR 'septic shock':ab,ti)

4806 records

**3. Cochrane Library**

#1 (BMI):ti,ab,kw OR (body mass index):ti,ab,kw OR (obese):ti,ab,kw OR (obesity):ti,ab,kw OR (overweight):ti,ab,kw

109329 records

#2 (sepsis):ti,ab,kw OR (septic shock):ti,ab,kw

14672 records

#3 #1 AND #2

372 records

**4. Web of Science**

((((((((((TI=(obese)) OR TI=(obesity)) OR TI=(overweight)) OR TI=(BMI)) OR TI=(body mass index)) OR AB=(obese)) OR AB=(obesity)) OR AB=(overweight)) OR AB=(BMI)) OR AB=(body mass index)) AND ((((TI=(septic shock) OR TI=(sepsis)) OR AB=(sepsis)) OR AB=(septic shock)) OR AB=(sepsis))

2250 records
