# Supplementary material for: Association of body mass index with mortality of sepsis or septic shock: an updated meta-analysis
Source: J Intensive Care. 2023 Jul 3;11:27. doi: 10.1186/s40560-023-00677-0 (PMC10316562; doi:10.1186/s40560-023-00677-0)
Supplement: Supplementary file 3 — Additional file 3. Newcastle-Ottawa Quality Assessment Scale. [file 40560_2023_677_MOESM3_ESM.docx]

| **Additional Table 1. Newcastle-Ottawa Quality Assessment Scale** |
| --- |
| **Case**-**Control Studies** |
| **Selection**  **(1) Is the case definition adequate?**  (a) yes, with independent validation ★  (b) yes, e.g., record linkage or based on self-reports  (c) no description  **(2) Representativeness of the cases**  (a) consecutive or obviously representative series of cases ★  (b) potential for selection biases or not stated  **(3) Selection of Controls**  (a) community controls ★  (b) hospital controls  (c) no description  **(4) Definition of Controls**  (a) no history of disease (endpoint) ★  (b) no description of source |
| **Comparability**  **(1) Comparability of cases and controls on the basis of the design or analysis**  (a) study controls for ____ (Select the most important factor.) ★  (b) study controls for any additional factor (These criteria could be modified to indicate specific control for a second important factor.) ★ |
| **Exposure**  **(1) Ascertainment of exposure**  (a) secure record (e.g., surgical records) ★  (b) structured interview where blind to case/control status ★  (c) interview not blinded to case/control status  (d) written self-report or medical record only  (e) no description  **(2) Same method of ascertainment for cases and controls**  (a) yes ★  (b) no  **(3) Non-Response rate**  (a) same rate for both groups ★  (b) non respondents described  (c) rate different and no designation |
| **Cohort Studies** |
| **Selection**  **(1) Representativeness of the exposed cohort**  (a) truly representative of the average ______ (describe) in the community ★  (b) somewhat representative of the average _____in the community ★  (c) selected group of users e.g., nurses, volunteers  (d) no description of the derivation of the cohort  **(2) Selection of the non-exposed cohort**  (a) drawn from the same community as the exposed cohort ★  (b) drawn from a different source  (c) no description of the derivation of the non-exposed cohort  **(3) Ascertainment of exposure**  (a) secure record (e.g., surgical records) ★  (b) structured interview ★  (c) written self-report  (d) no description  **(4) Demonstration that outcome of interest was not present at start of study**  (a) yes ★  (b) no |
| **Comparability**  **(1) Comparability of cohorts on the basis of the design or analysis**  (a) study controls for ______ (select the most important factor) ★  (b) study controls for any additional factor (These criteria could be modified to indicate specific control for a second important factor.) ★ |
| **Outcome**  **(1) Assessment of outcome**  (a) independent blind assessment ★  (b) record linkage ★  (c) self-report  (d) no description  **(2) Was follow**-**up long enough for outcomes to occur**  (a) yes (select an adequate follow up period for outcome of interest) ★  (b) no  **(3) Adequacy of follow up of cohorts**  (a) complete follow up - all subjects accounted for ★  (b) subjects lost to follow up unlikely to introduce bias - small number lost > ____ % (select an adequate %) follow up, or description provided of those lost) ★  (c) follow up rate < ____% (select an adequate %) and no description of those lost  (d) no statement |

Note:

- means being awarded 1 star.

A study can be awarded a maximum of 1 star for each numbered item within the Selection and Exposure categories. A maximum of 2 stars can be given for Comparability.

Good quality: 3 or 4 stars in selection domain AND 1 or 2 stars in comparability domain AND 2 or 3 stars in outcome/exposure domain.

Fair quality: 2 stars in selection domain AND 1 or 2 stars in comparability domain AND 2 or 3 stars in outcome/exposure domain.

Poor quality: 0 or 1 star in selection domain OR 0 star in comparability domain OR 0 or 1 star in outcome/exposure domain.
