# Supplementary material for: Association of body mass index with mortality of sepsis or septic shock: an updated meta-analysis
Source: J Intensive Care. 2023 Jul 3;11:27. doi: 10.1186/s40560-023-00677-0 (PMC10316562; doi:10.1186/s40560-023-00677-0)
Supplement: Supplementary file 4 — Additional file 4. The Newcastle-Ottawa Quality Assessment Scale of Included Case-Control or Cohort Studies. [file 40560_2023_677_MOESM4_ESM.docx]

**Additional Table S2. The Newcastle-Ottawa Quality Assessment Scale of Included Case**-**Control or Cohort Studies**

| **Cohort Studies** | | | | | | | | | |
| --- | --- | --- | --- | --- | --- | --- | --- | --- | --- |
| Study | Representativeness of the exposed cohort | Selection of the non-exposed cohort | Ascertainment of exposure | Demonstration that outcome of interest was not present at start of study | Comparability of cohorts on the basis of the design or analysis | Assessment of outcome | Was follow-up long enough for outcomes to occur | Adequacy of follow up of cohorts | Quality Score |
| Wurzinger et al.[1] | ★ | ★ | ★ | ★ | ★★ | ★ | ★ | ★ | 9 |
| Pepper et al.[2] | ★ | ★ | ★ | ★ | ★ | ★ | ★ | 0 | 7 |
| Tay-Lasso et al.[3] | ★ | ★ | ★ | ★ | ★★ | ★ | ★ | 0 | 8 |
| Kuperman et al.[4] | ★ | ★ | ★ | ★ | ★ | ★ | ★ | ★ | 8 |
| Prescott et al.[5] | ★ | ★ | 0 | ★ | ★★ | ★ | ★ | ★ | 8 |
| Sakr et al.[6] | ★ | ★ | 0 | ★ | ★★ | ★ | ★ | ★ | 8 |
| Lin et al.[7] | ★ | ★ | ★ | ★ | ★★ | ★ | ★ | 0 | 8 |
| Danninger et al.[8] | ★ | ★ | ★ | ★ | ★★ | ★ | ★ | ★ | 9 |
| Gaulton et al.[9] | ★ | ★ | ★ | ★ | ★★ | ★ | ★ | ★ | 9 |
| Gaulton et al.[10] | ★ | ★ | ★ | ★ | ★★ | ★ | ★ | ★ | 9 |
| Arabi et al.[11] | ★ | ★ | ★ | ★ | ★★ | ★ | ★ | 0 | 8 |
| Chae et al.[12] | ★ | ★ | ★ | ★ | ★★ | ★ | ★ | ★ | 9 |
| Li et al.[13] | ★ | ★ | ★ | ★ | ★★ | ★ | ★ | 0 | 8 |
| Juarez et al.[14] | ★ | ★ | ★ | ★ | ★ | ★ | ★ | ★ | 8 |
| Yeo et al.[15] | ★ | ★ | ★ | ★ | ★★ | ★ | ★ | ★ | 9 |

Note:

★ means being awarded 1 star.

★★ means being awarded 2 stars.

**Reference**

1. Wurzinger B, Dünser MW, Wohlmuth C, et al. The association between body-mass index and patient outcome in septic shock: a retrospective cohort study. *Wiener klinische Wochenschrift*. Jan 2010;122(1-2):31-6.

2. Pepper DJ, Demirkale CY, Sun J, et al. Does Obesity Protect Against Death in Sepsis? A Retrospective Cohort Study of 55,038 Adult Patients. *Critical care medicine*. May 2019;47(5):643-650.

3. Tay-Lasso E, Grigorian A, Lekawa M, et al. Obesity Does Not Increase Risk for Mortality in Severe Sepsis Trauma Patients. *The American surgeon*. Mar 2 2022:31348221078986.

4. Kuperman EF, Showalter JW, Lehman EB, Leib AE, Kraschnewski JL. The impact of obesity on sepsis mortality: a retrospective review. *BMC infectious diseases*. Aug 16 2013;13:377.

5. Prescott HC, Chang VW, O'Brien JM, Jr., Langa KM, Iwashyna TJ. Obesity and 1-year outcomes in older Americans with severe sepsis. *Critical care medicine*. Aug 2014;42(8):1766-74.

6. Sakr Y, Madl C, Filipescu D, et al. Obesity is associated with increased morbidity but not mortality in critically ill patients. *Intensive care medicine*. Nov 2008;34(11):1999-2009.

7. Lin S, Ge S, He W, Zeng M. Association between Body Mass Index and Short-Term Clinical Outcomes in Critically Ill Patients with Sepsis: A Real-World Study. *BioMed research international*. 2020;2020:5781913.

8. Danninger T, Rezar R, Mamandipoor B, et al. Underweight but not overweight is associated with excess mortality in septic ICU patients. *Wiener klinische Wochenschrift*. Feb 2022;134(3-4):139-147.

9. Gaulton TG, Marshall MacNabb C, Mikkelsen ME, et al. A retrospective cohort study examining the association between body mass index and mortality in severe sepsis. *Internal and emergency medicine*. Jun 2015;10(4):471-9.

10. Gaulton TG, Weiner MG, Morales KH, Gaieski DF, Mehta J, Lautenbach E. The effect of obesity on clinical outcomes in presumed sepsis: a retrospective cohort study. *Internal and emergency medicine*. Mar 2014;9(2):213-21.

11. Arabi YM, Dara SI, Tamim HM, et al. Clinical characteristics, sepsis interventions and outcomes in the obese patients with septic shock: an international multicenter cohort study. *Critical care (London, England)*. Apr 17 2013;17(2):R72.

12. Chae MK CD, Shin TG, Jeon K, Suh GY, Sim MS, Song KJ, Jeong YK, Jo IJ. Body Mass Index and Outcomes in Patients with Severe Sepsis or Septic Shock. *Korean Journal of Critical Care Medicine*. 2013;28(4):266-271.

13. Li S, Hu X, Xu J, et al. Increased body mass index linked to greater short- and long-term survival in sepsis patients: A retrospective analysis of a large clinical database. *International journal of infectious diseases : IJID : official publication of the International Society for Infectious Diseases*. Oct 2019;87:109-116.

14. Juarez E EH, Lear M, Sanchez A, Yang S, Nugent K. The association between body mass index and outcomes in patient with sepsis and acute respiratory failure. *The Southwest Respiratory and Critical Care Chronicles*. 2019;7(31):13-23.

15. Yeo HJ, Kim TH, Jang JH, et al. Obesity Paradox and Functional Outcomes in Sepsis: A Multicenter Prospective Study. *Critical care medicine*. 2023;51(6):742-752
