# Supplementary material for: Association of body mass index with mortality of sepsis or septic shock: an updated meta-analysis
Source: J Intensive Care. 2023 Jul 3;11:27. doi: 10.1186/s40560-023-00677-0 (PMC10316562; doi:10.1186/s40560-023-00677-0)
Supplement: Supplementary file 5 — Additional file 5. Supplementary figures. [file 40560_2023_677_MOESM5_ESM.docx]

Additional Figure 1A. Individual and pooled results of the association of underweight BMIs with mortality of sepsis in patients > 50 years.


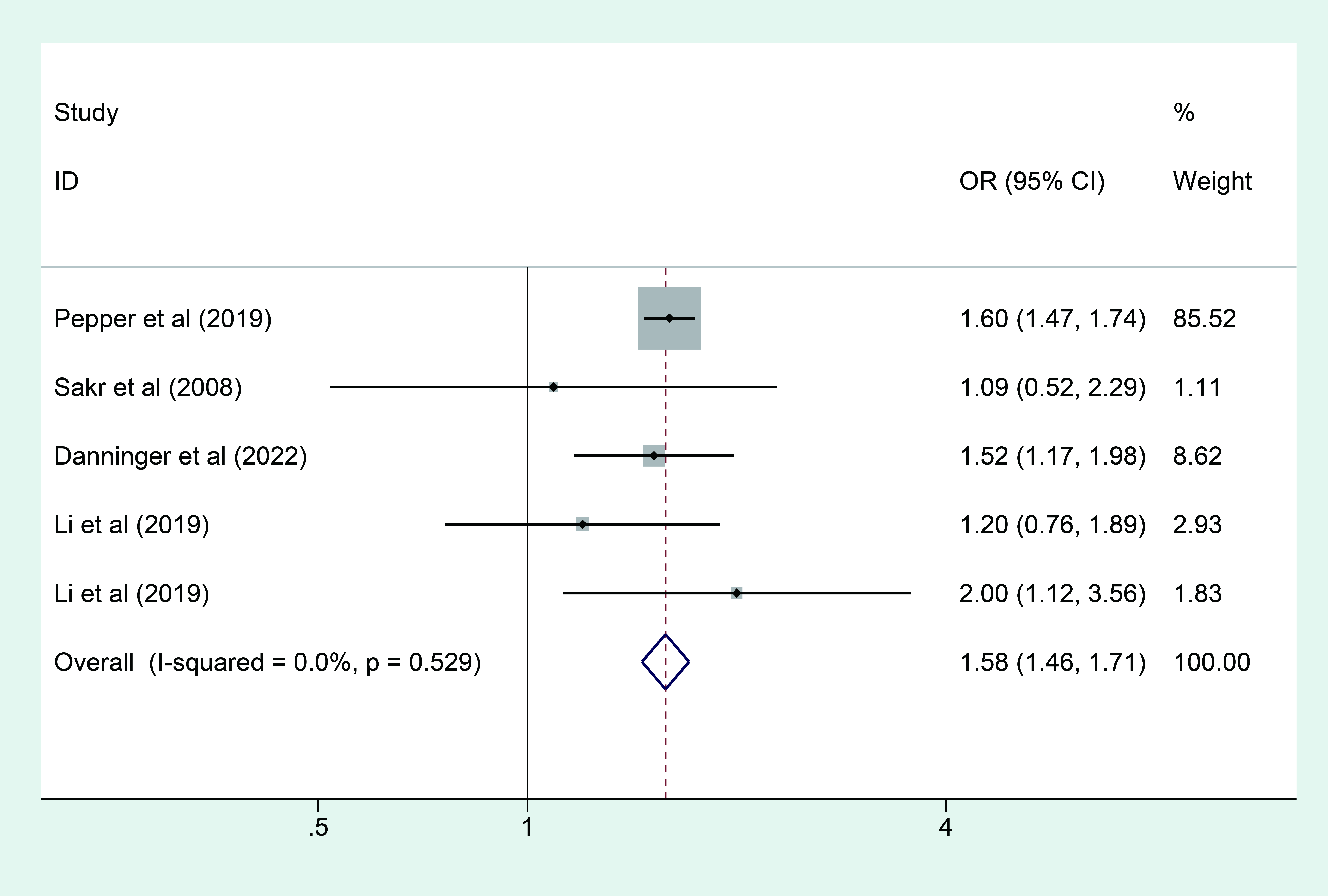


Additional Figure 1B. Individual and pooled results of the association of underweight BMIs with mortality of sepsis in patients ≤ 50 years.

A
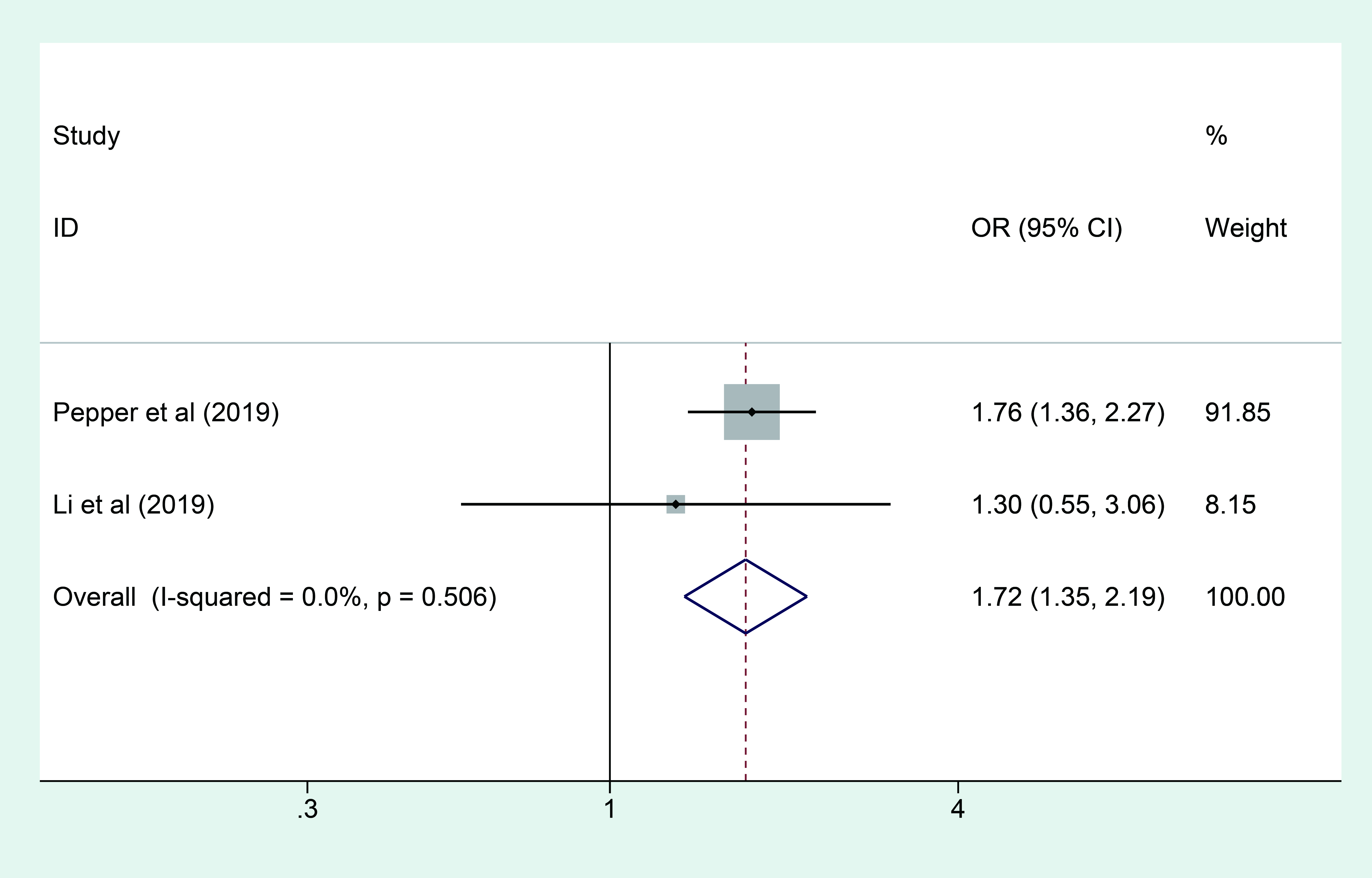


Additional Figure 1C. Individual and pooled results of the association of overweight BMIs with mortality of sepsis in patients > 50 years.


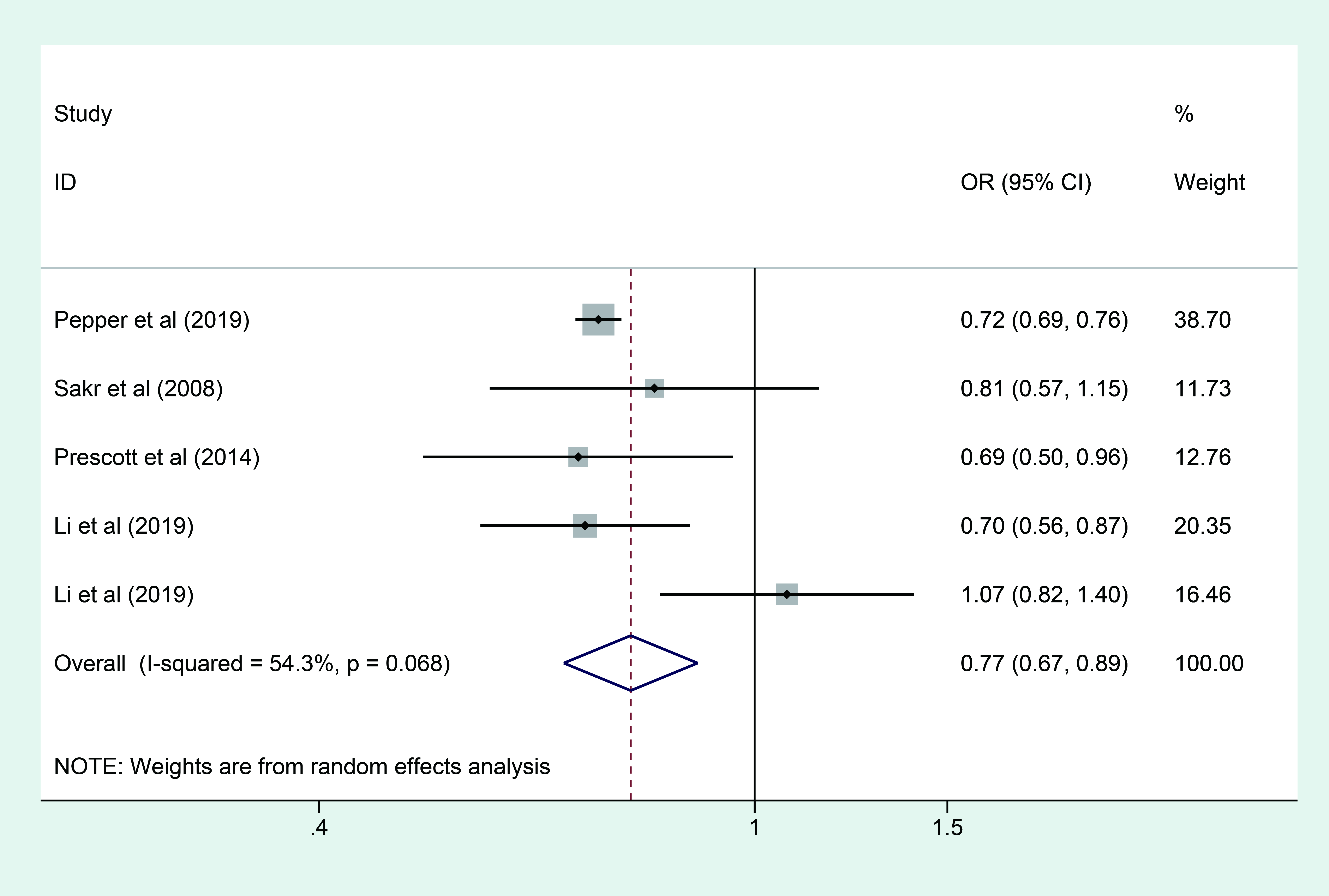


Additional Figure 1D. Individual and pooled results of the association of overweight BMIs with mortality of sepsis in patients ≤ 50 years.


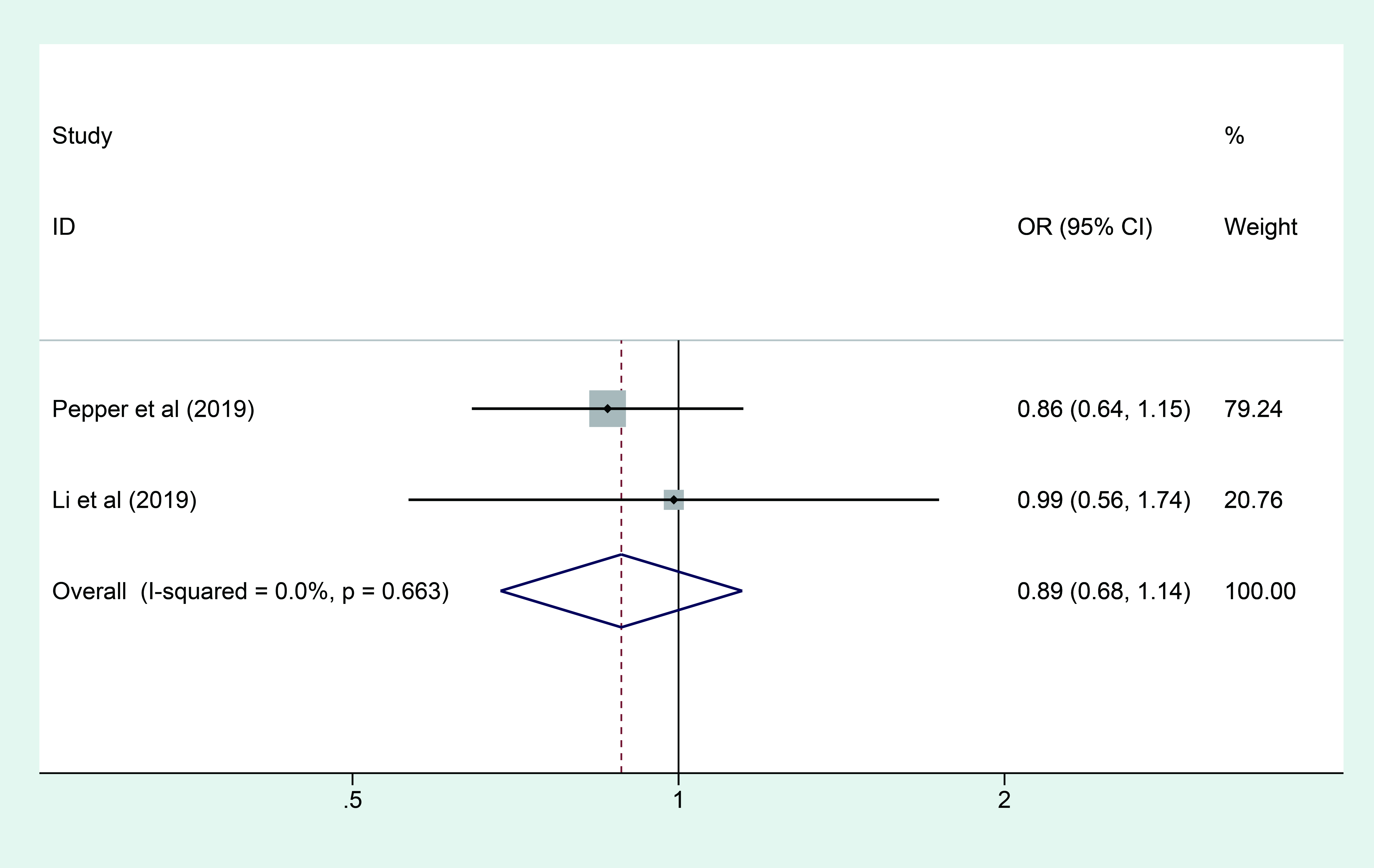


Additional Figure 1E. Individual and pooled results of the association of obese BMIs with mortality of sepsis in patients > 50 years.


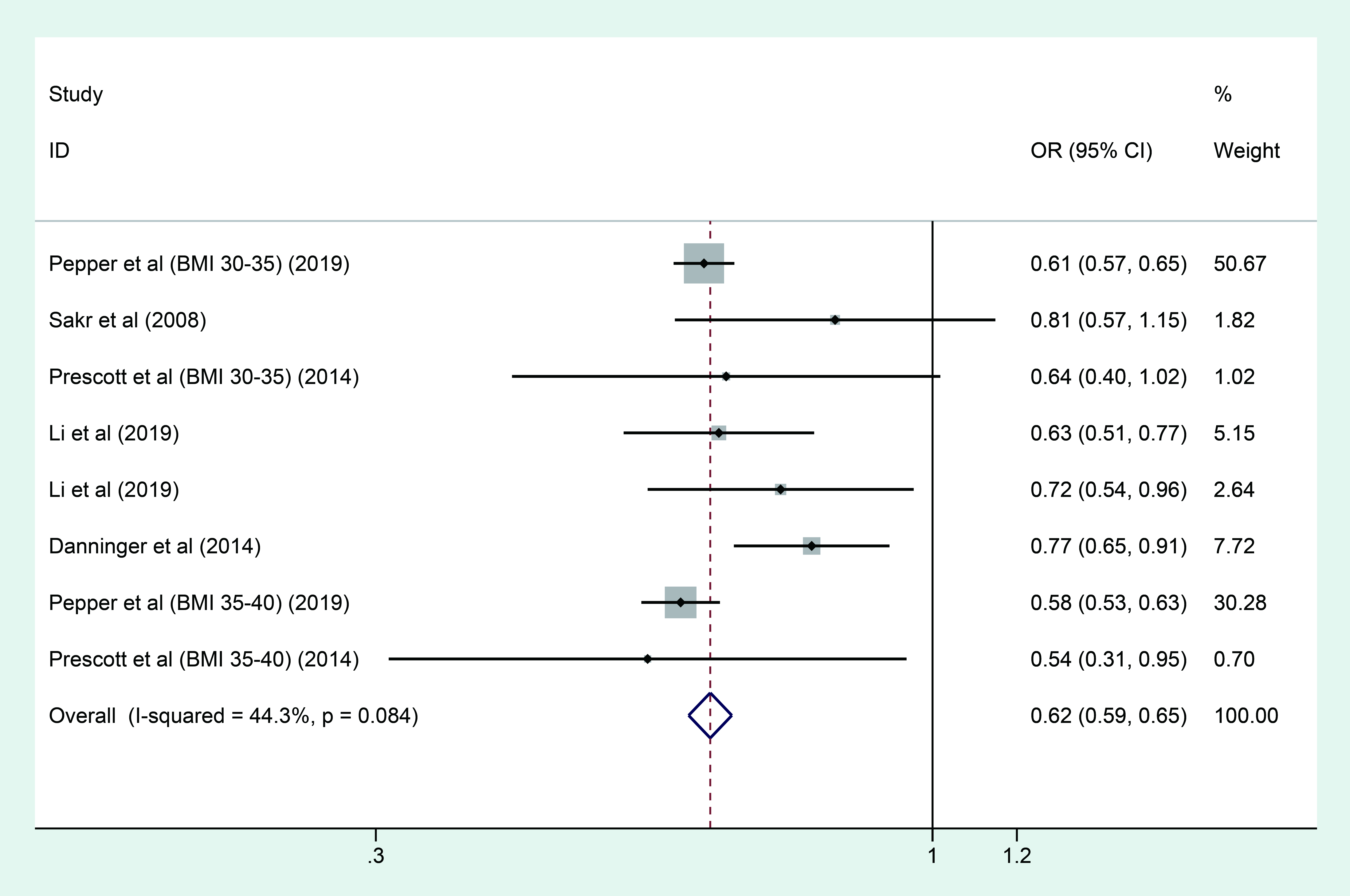


Additional Figure 1F. Individual and pooled results of the association of obese BMIs with mortality of sepsis in patients ≤ 50 years.


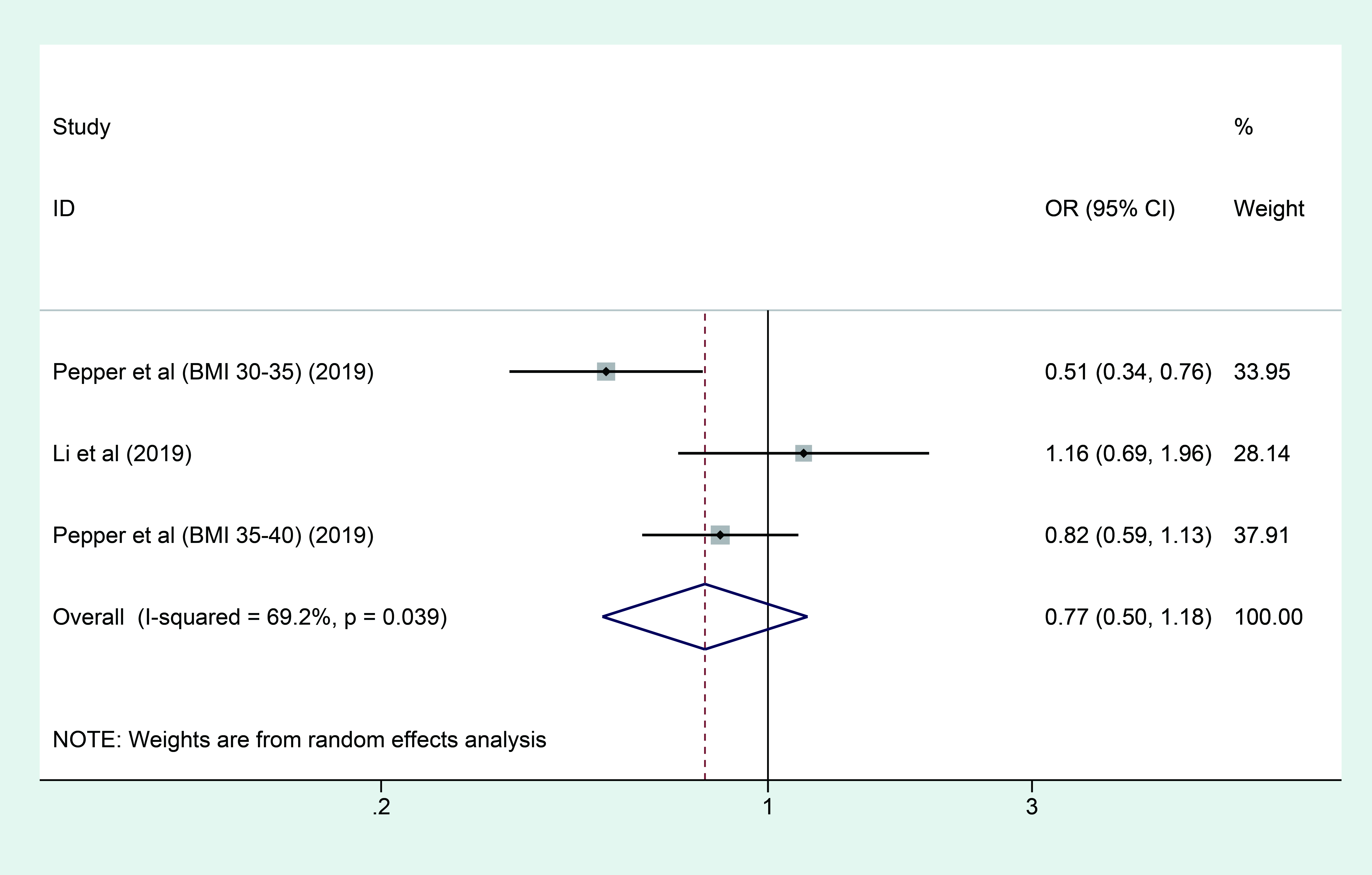


Additional Figure 2A. Individual and pooled results of the association of underweight BMIs with mortality of sepsis in retrospective studies.





Additional Figure 2B. Individual and pooled results of the association of underweight BMIs with mortality of sepsis in prospective studies.


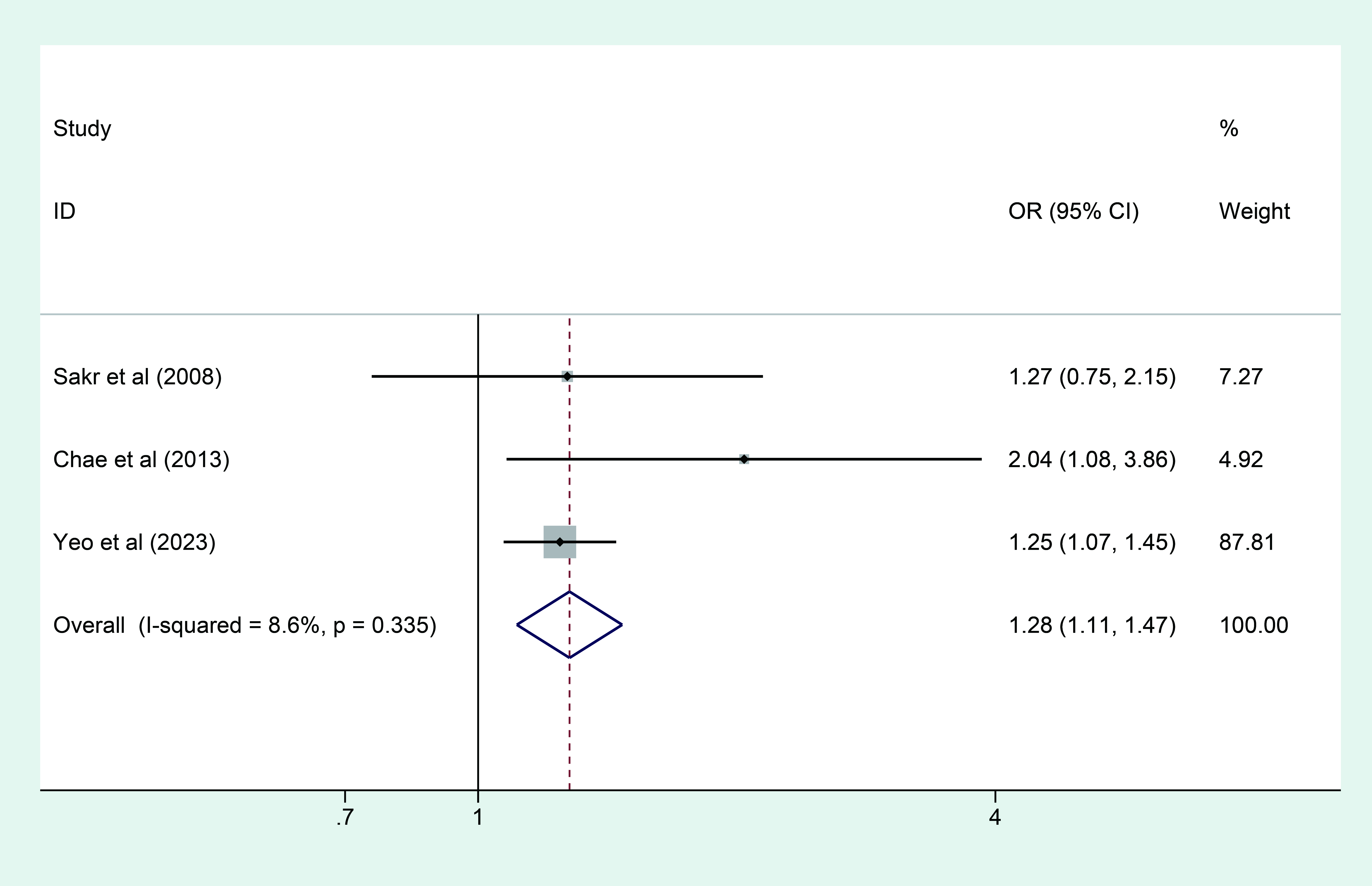


Additional Figure 2C. Individual and pooled results of the association of overweight BMIs with mortality of sepsis in retrospective studies.





Additional Figure 2D. Individual and pooled results of the association of overweight BMIs with mortality of sepsis in prospective studies.


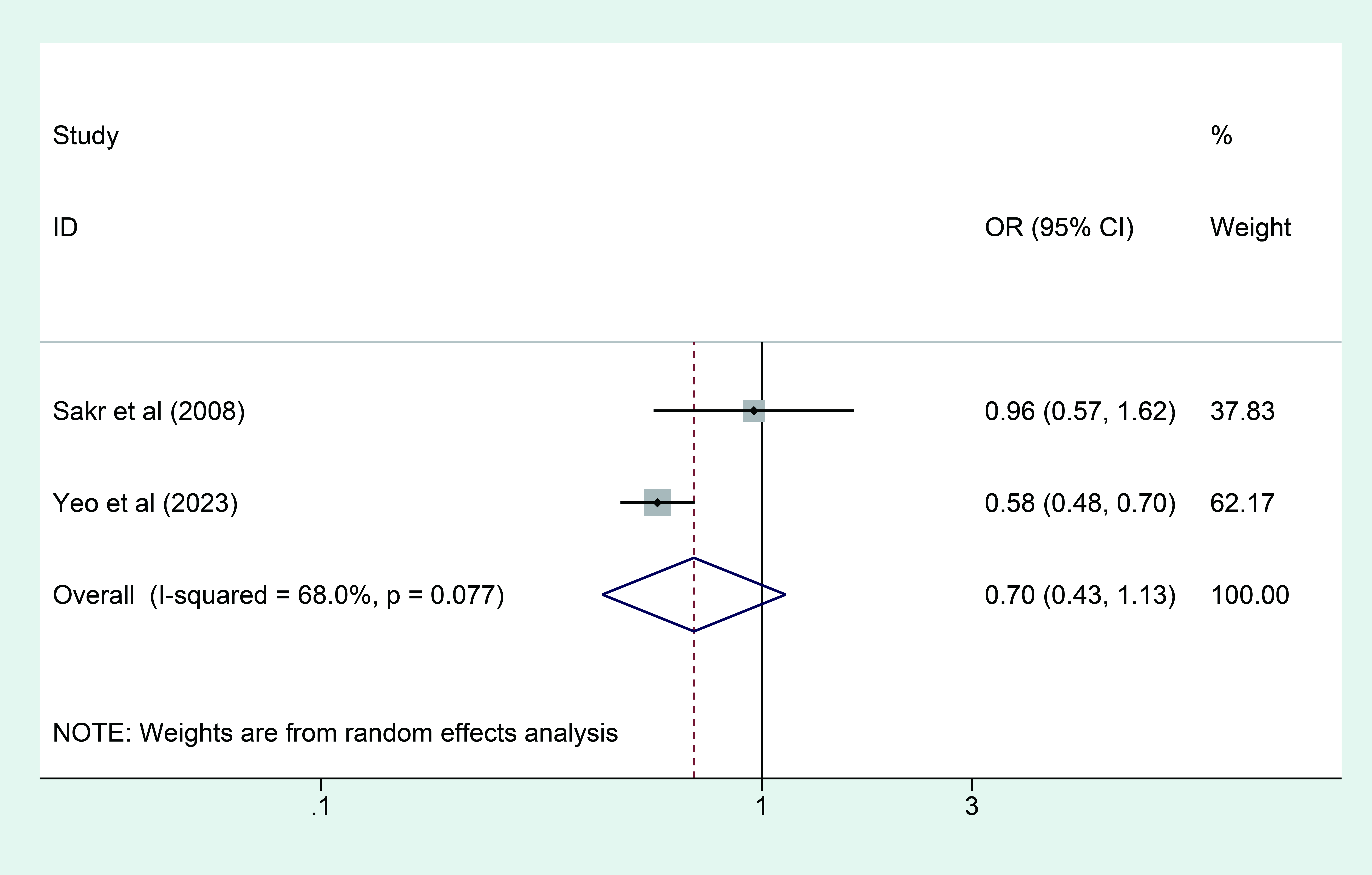


Additional Figure 2E. Individual and pooled results of the association of obese BMIs with mortality of sepsis in retrospective studies.





Additional Figure 2F. Individual and pooled results of the association of obese BMIs with mortality of sepsis in prospective studies.


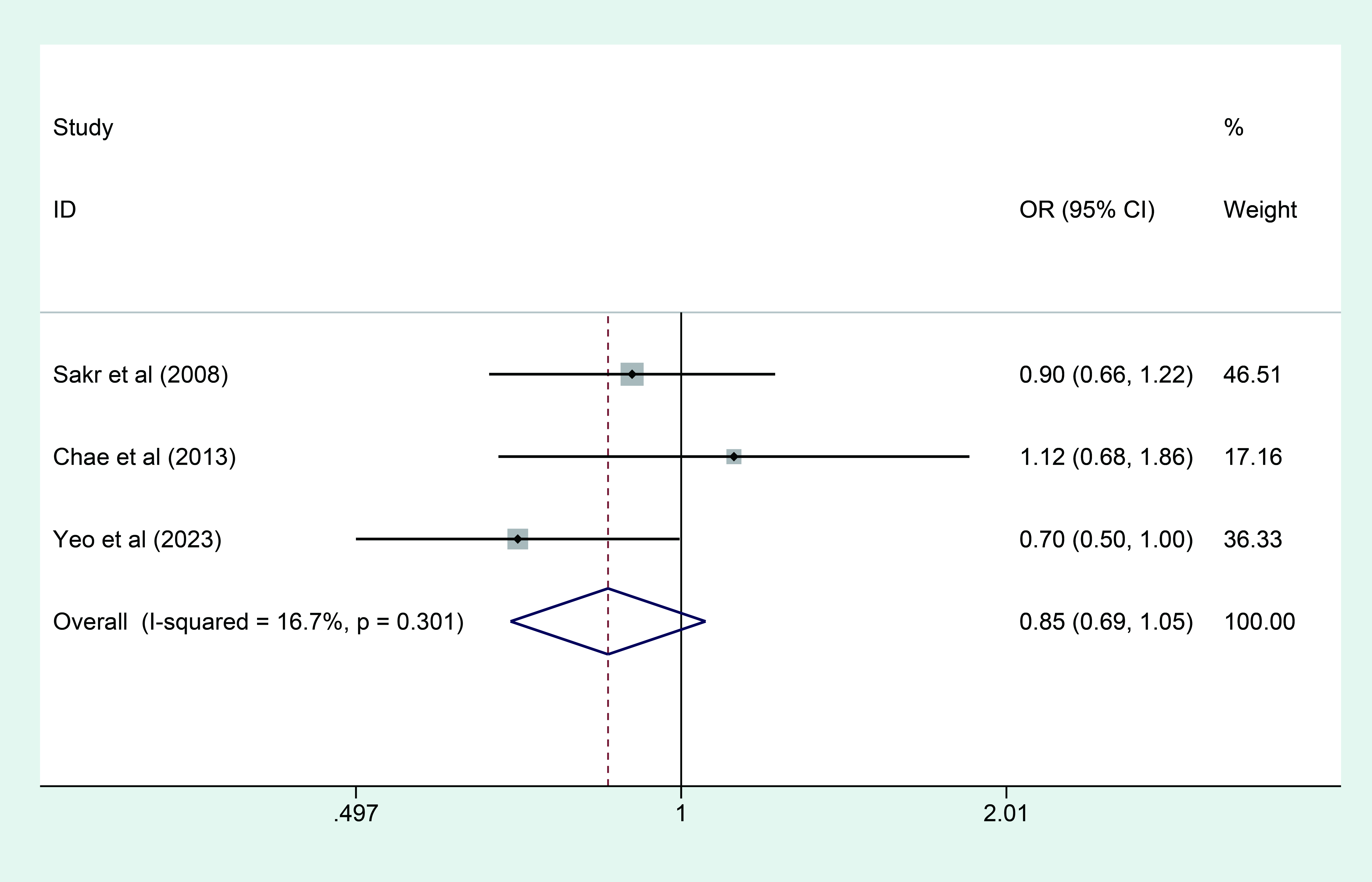


Additional Figure 3A. Individual and pooled results of the association of underweight BMIs with mortality of sepsis in studies where diagnoses were based on Sepsis 1.0 or 2.0 criteria.


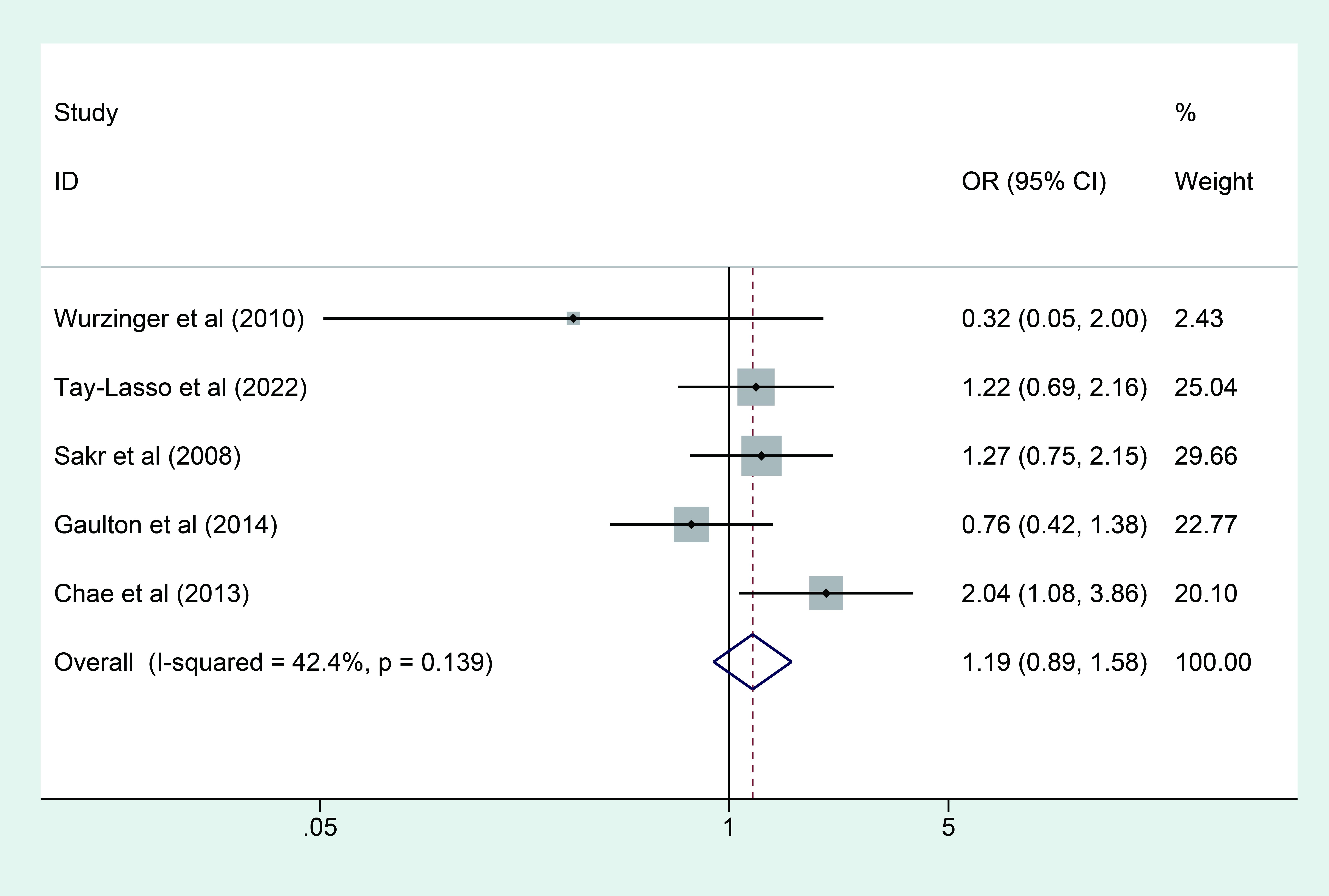


Additional Figure 3B. Individual and pooled results of the association of underweight BMIs with mortality of sepsis in studies where diagnoses were based on Sepsis 3.0 criteria.


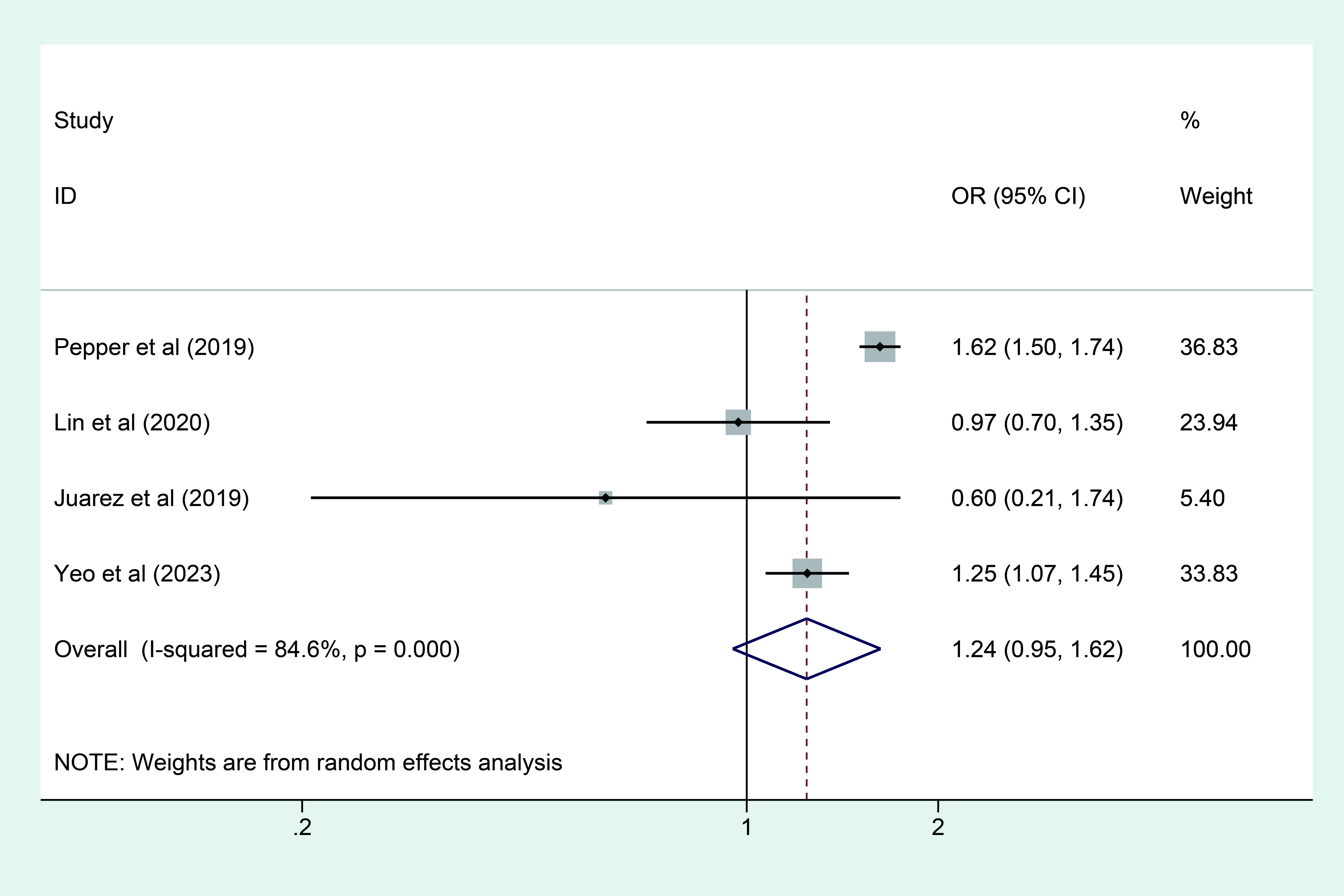


Additional Figure 3C. Individual and pooled results of the association of overweight BMIs with mortality of sepsis in studies where diagnoses were based on Sepsis 1.0 or 2.0 criteria.


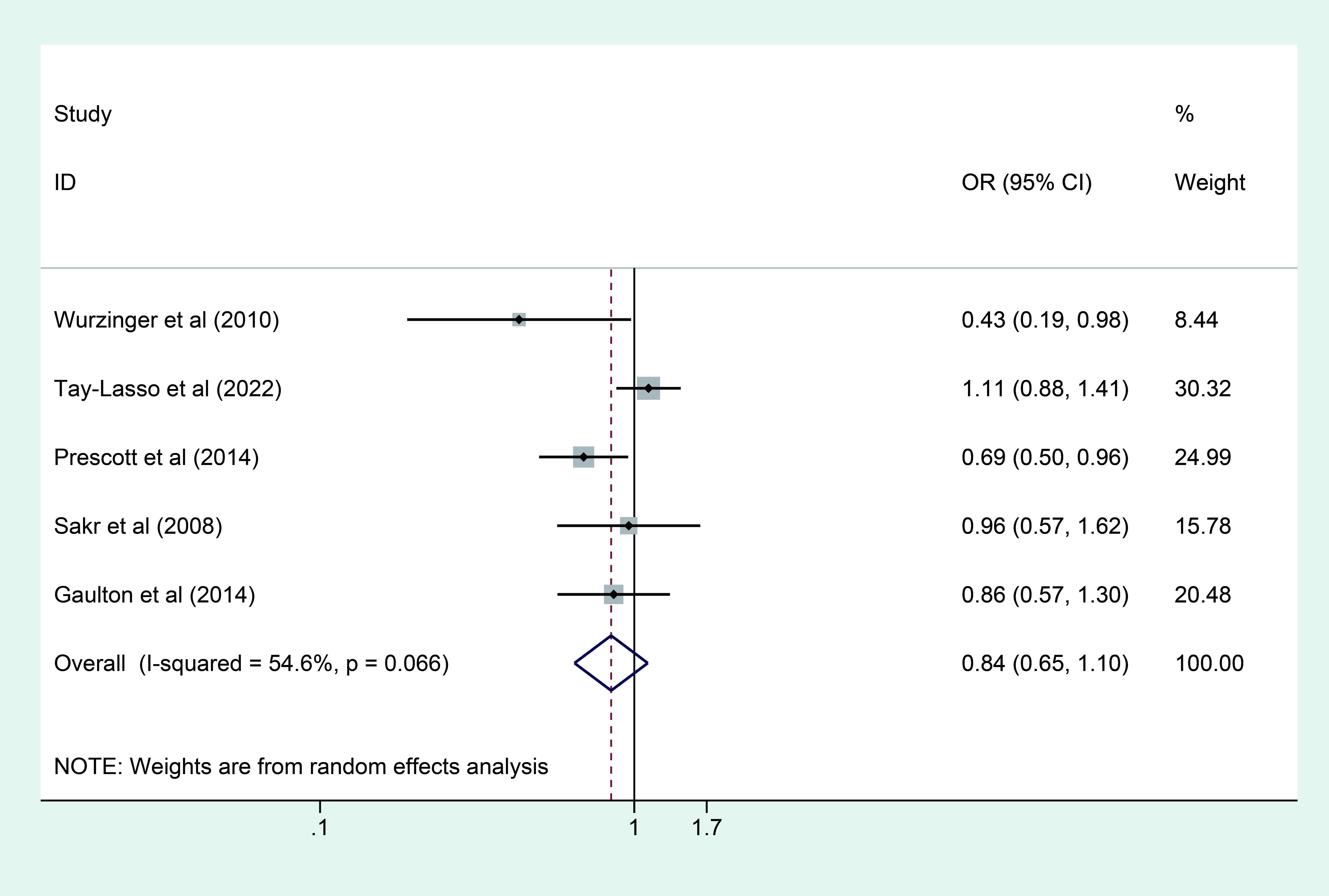


Additional Figure 3D. Individual and pooled results of the association of overweight BMIs with mortality of sepsis in studies where diagnoses were based on Sepsis 3.0 criteria.


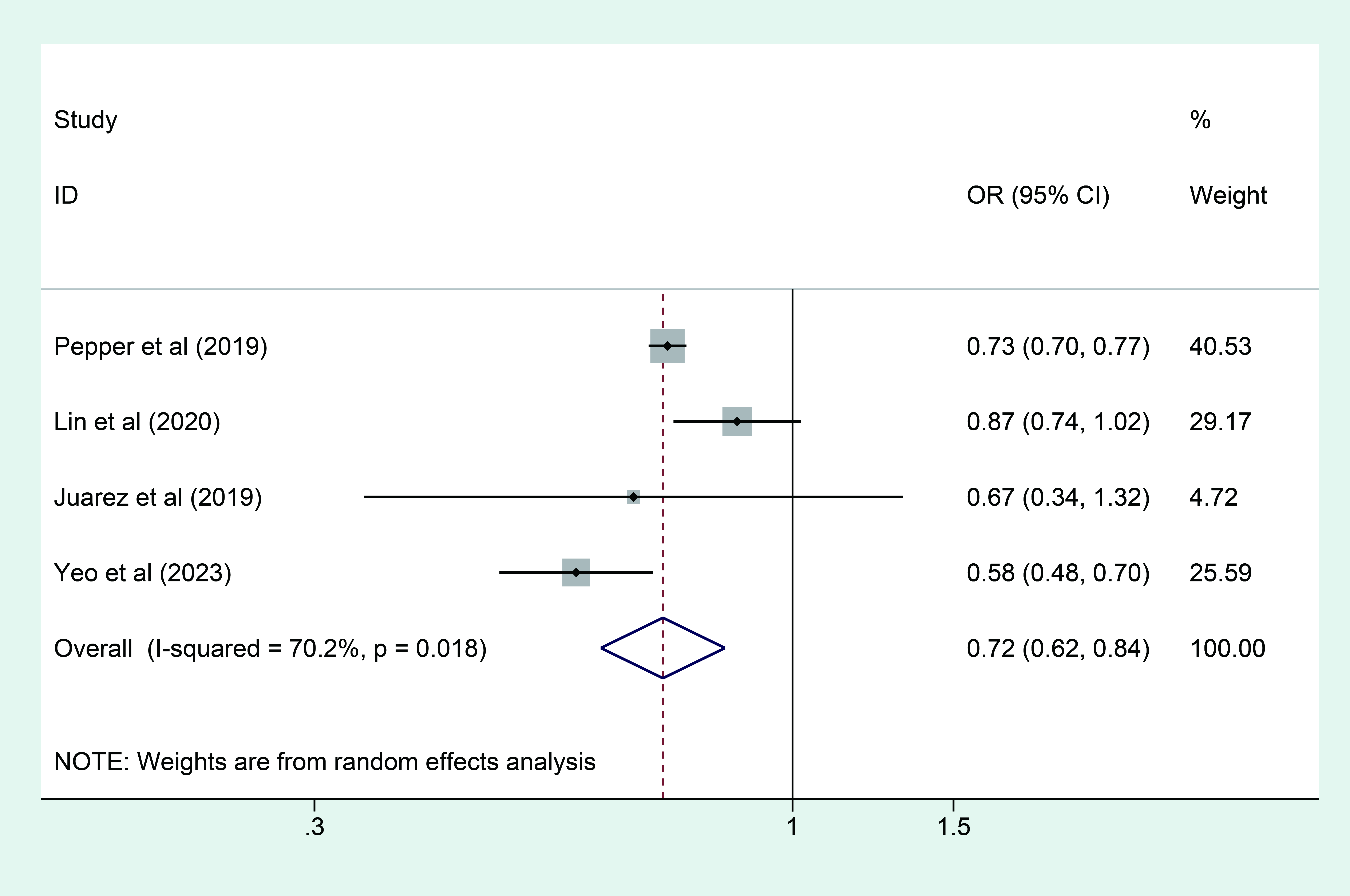


Additional Figure 3E. Individual and pooled results of the association of obese BMIs with mortality of sepsis in studies where diagnoses were based on Sepsis 1.0 or 2.0 criteria.





Additional Figure 3F. Individual and pooled results of the association of obese BMIs with mortality of sepsis in studies where diagnoses were based on Sepsis 3.0 criteria.


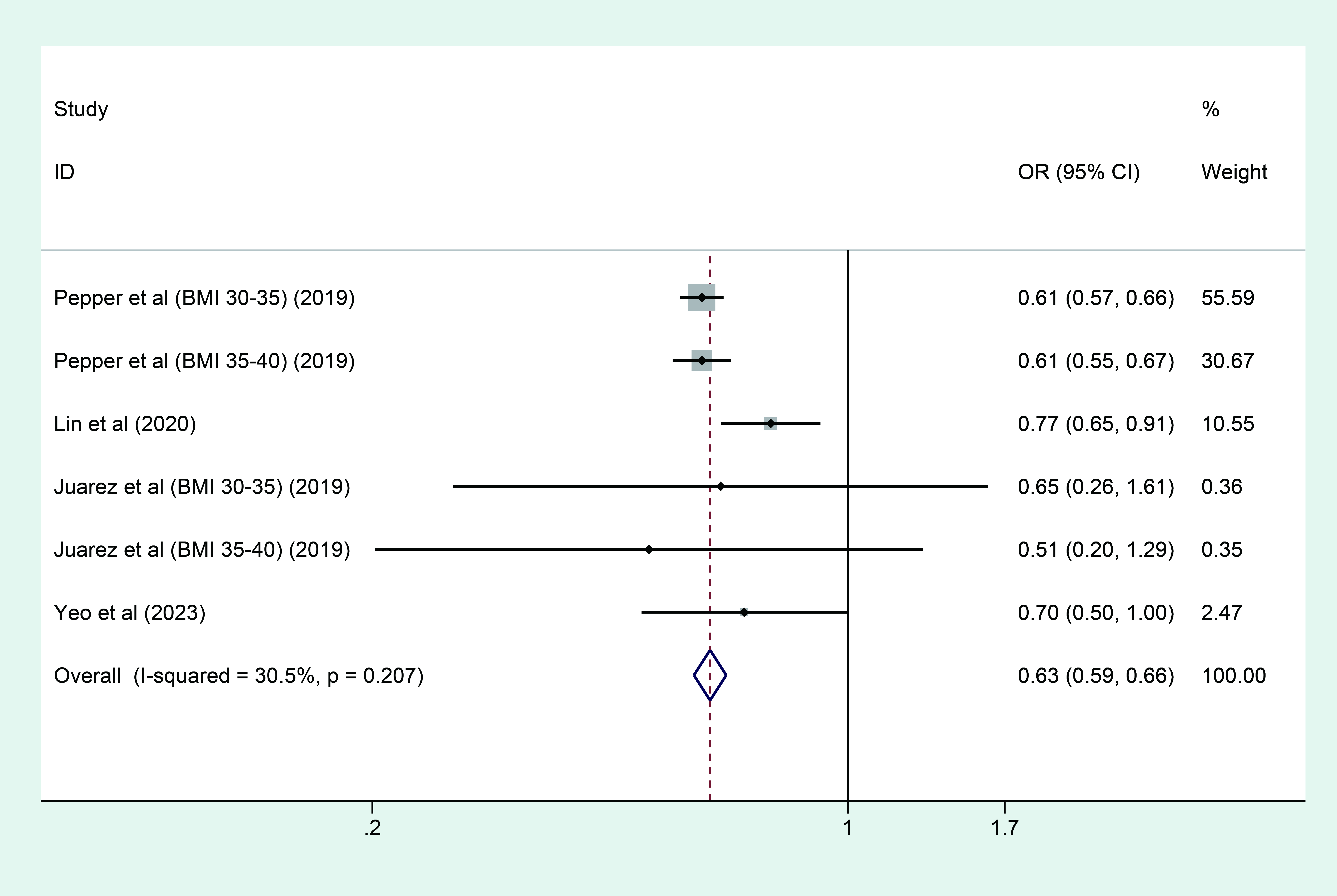


Additional Figure 4A. Individual and pooled results of the association of underweight BMIs with mortality in patients diagnosed with sepsis.





Additional Figure 4B. Individual and pooled results of the association of underweight BMIs with mortality in patients diagnosed with severe sepsis or septic shock.


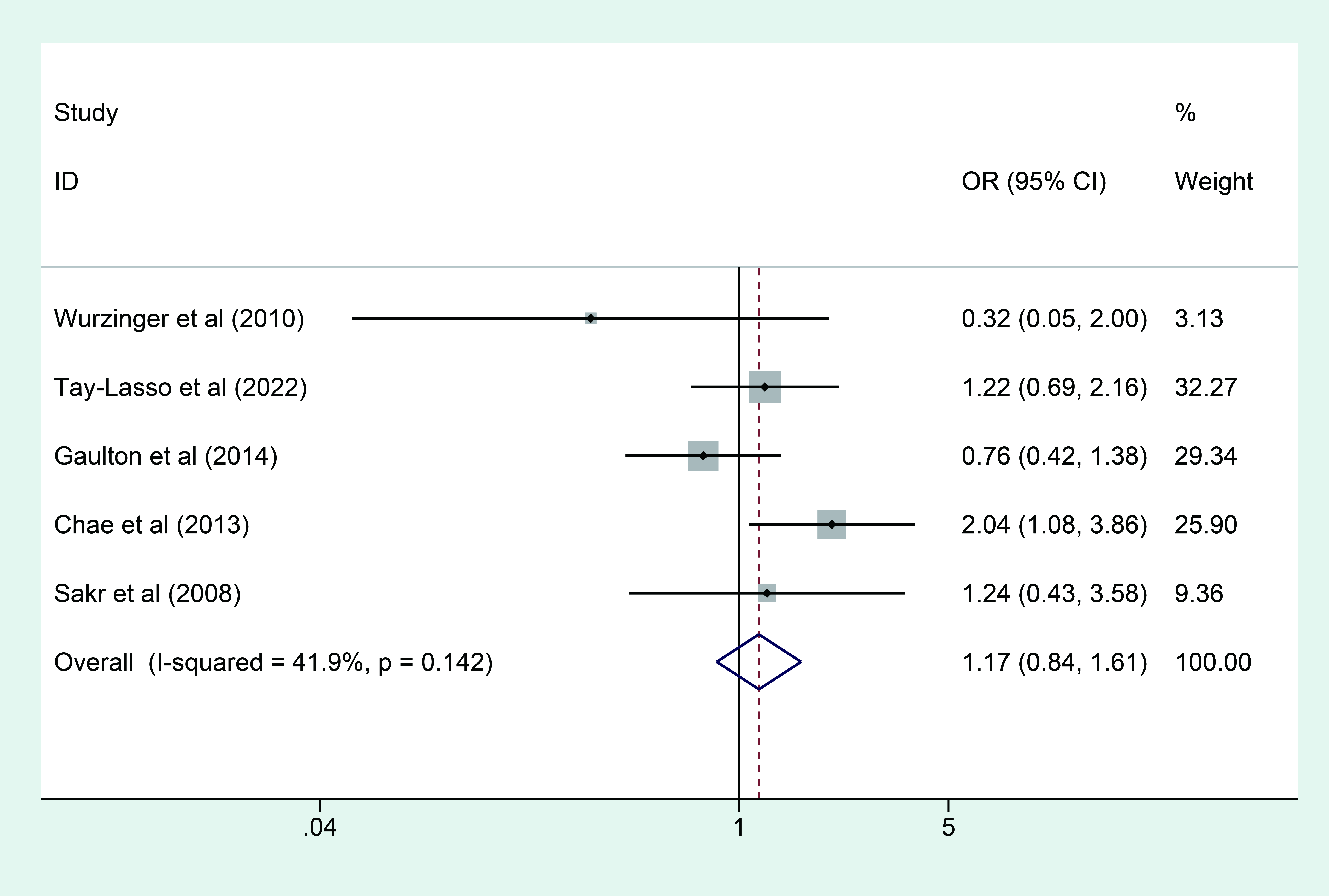


Additional Figure 4C. Individual and pooled results of the association of overweight BMIs with mortality in patients diagnosed with sepsis.





Additional Figure 4D. Individual and pooled results of the association of overweight BMIs with mortality in patients diagnosed with severe sepsis or septic shock.


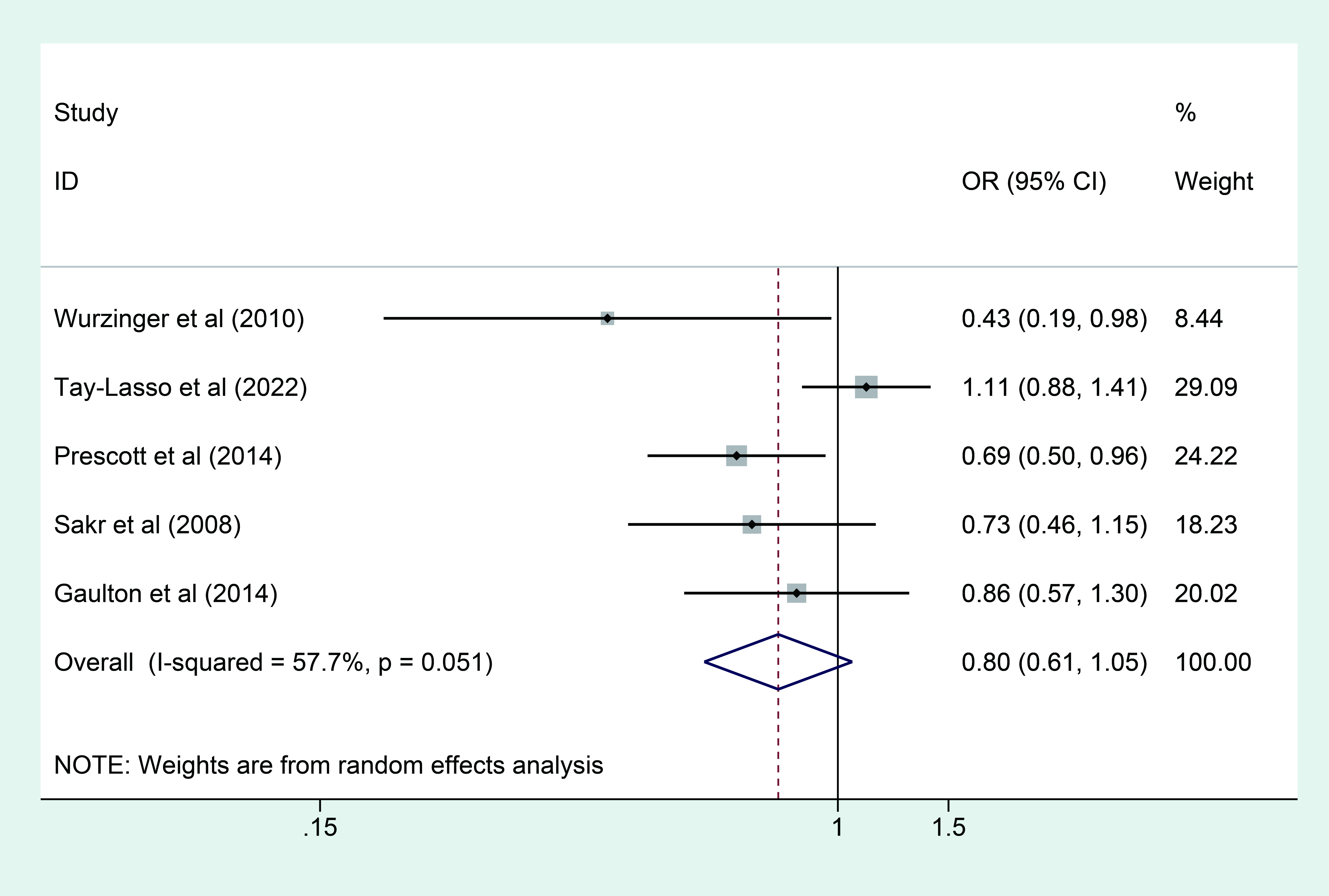


Additional Figure 4E. Individual and pooled results of the association of obese BMIs with mortality in patients diagnosed with sepsis.





Additional Figure 4F. Individual and pooled results of the association of obese BMIs with mortality in patients diagnosed with severe sepsis or septic shock.
